# Deep-learning prediction of gene expression from personal genomes

**DOI:** 10.1186/s13059-025-03926-7

**Published:** 2026-01-06

**Authors:** Shiron Drusinsky, Sean Whalen, Katherine S. Pollard

**Affiliations:** 1 Gladstone Institutes, San Francisco, CA 94158 USA; 2 Biomedical Informatics Graduate Program, University of California, San Francisco, San Francisco, CA 94158 USA; 3 Chan Zuckerberg Biohub San Francisco, San Francisco, CA 94158 USA; 4 Department of Epidemiology and Biostatistics, University of California, San Francisco, San Francisco, CA 94158 USA

**Keywords:** Deep learning, Genomics, Gene expression, Genetic variants, Regulatory elements

## Abstract

**Background:**

Models that predict gene expression levels from DNA sequence struggle to predict differences between individuals when given their personal genome sequences. These models are generally trained on reference genome sequences, and thus have never observed examples of genetic variation at any locus during training, which may explain their lack of generalizability to personal genome sequences that do contain variation.

**Results:**

We utilize fine-tuning with personal genomes and matched tissue-specific gene expression values to develop Variformer, a deep sequence-based neural network. Across held-out people, Variformer predicts expression with accuracy that approaches the cis-heritability of most genes and prioritizes genetic variants across the allele frequency spectrum that are enriched for motif disruption and other functional annotations. We highlight how Variformer fails to generalize to unseen genes.

**Conclusions:**

Our work suggests that fine-tuning with personal genomes corrects previously reported shortcomings of gene expression prediction across unseen individuals, but does not learn a regulatory grammar that generalizes to unseen loci. Fine-tuned deep expression models thus share similar performance and limitations of state-of-the-art linear models, highlighting a gap for the field.

**Supplementary Information:**

The online version contains supplementary material available at 10.1186/s13059-025-03926-7.

## Background

Models that predict RNA levels from DNA sequences show tremendous promise for decoding tissue-specific gene regulatory mechanisms [1–5], revealing the genetic architecture of traits [6–10], and interpreting noncoding genetic variation [10, 11]. Existing methods take two complementary approaches to predicting gene expression: 1) associating expression with linear combinations of individual genetic variants without flanking non-variable sequence context [12, 13], or 2) learning genome-wide sequence-to-expression rules with neural networks [11, 14, 15]. These two strategies differ not only in data inputs and model architectures, but also in training strategies. State-of-the-art linear models [6, 8, 9, 12] typically use penalized regression to identify common variants that predict expression of a single gene across a sample of genotyped individuals with RNA-seq data from one or more tissues/cell types (training across individuals on single genes). On the other hand, deep learning models tend to use a single reference genome per species to predict genome-wide epigenetic and expression data from many tissues/cell types (training across loci using a reference genome) [7, 11, 14, 15].

Recent work has highlighted limitations of both existing strategies to gene expression prediction [16–20]. Linear models can explain much of expression cis-heritability [6, 21]. However, they 1) are not designed to make predictions for new genes or variants, 2) largely ignore the contributions of rare variants, 3) identify expression quantitative trait locus (eQTL) variants that frequently do not co-localize with trait-associated variants, and 4) require additional fine-mapping and experiments (e.g., epigenetic data) to pinpoint causal alleles [19, 20, 22]. Hence, linear models are unable to generalize beyond common variants with large effect sizes that are seen during training. In contrast, deep learning models can make accurate predictions at held-out genomic loci for the data sets on which they were trained. Feature attribution methods have been applied to deep learning models, showing promise for nominating causal alleles including tissue-specifying and disease variants [2, 5, 10, 11, 23]. However, existing neural network models cannot reliably explain inter-individual expression differences. They also struggle to predict eQTL direction and rely primarily on promoter variants [16, 18].

Before concluding that neural networks are unable to model inter-individual expression variation, we thought it would be important to apply cross-individual training and evaluation to cohorts of hundreds of individuals where linear models have been successful. Previous work used far fewer individuals and did not evaluate across them [7, 24]. To address this we developed Variformer, a fine-tuning strategy that implements cross-individual training and evaluation of sequence-to-expression neural network models. Briefly, we modified the Enformer architecture [15] by replacing the output head with one that predicts tissue-specific gene expression as a scalar value rather than a genomic track and fine-tuned the model using a custom loss function (Methods). We hypothesized that Variformer would retain Enformer’s knowledge of regulatory grammar while gaining the ability to capture expression cis-heritability.

## Results

### Fine-tuning on personal genomes improves predicted expression differences between unseen individuals, but not for unseen genes

To combine deep learning from genomic context with signals from genetic variants that correlate with inter-individual expression differences, we trained Variformer models and compared them to Enformer without fine-tuning and to elastic nets, a representative linear modeling framework that performs well at predicting expression variation [6, 16, 18] (Methods). We obtained paired whole-genome sequencing (WGS) and bulk RNA-seq data from the GTEx study [12], focusing initially on “Whole Blood'' samples because of the large sample size (*n* = 670). We selected ~ 300 genes from Enformer’s train set with a range of cis-heritabilities. For each person and gene we generated a 49-kilobase (kb) personalized genome sequence centered at the TSS, including all single-nucleotide variants (SNVs), and paired it with the same person’s normalized blood gene expression value. For each gene, we trained three elastic net and Variformer model replicates, matching the genomic window and how we varied individuals used for training and evaluation, enabling us to assess the mean and range of performance. While training Enformer from scratch can take weeks on multiple GPUs, fine-tuning takes only ~ 30 min per gene on a single GPU.

**Fig. 1:**
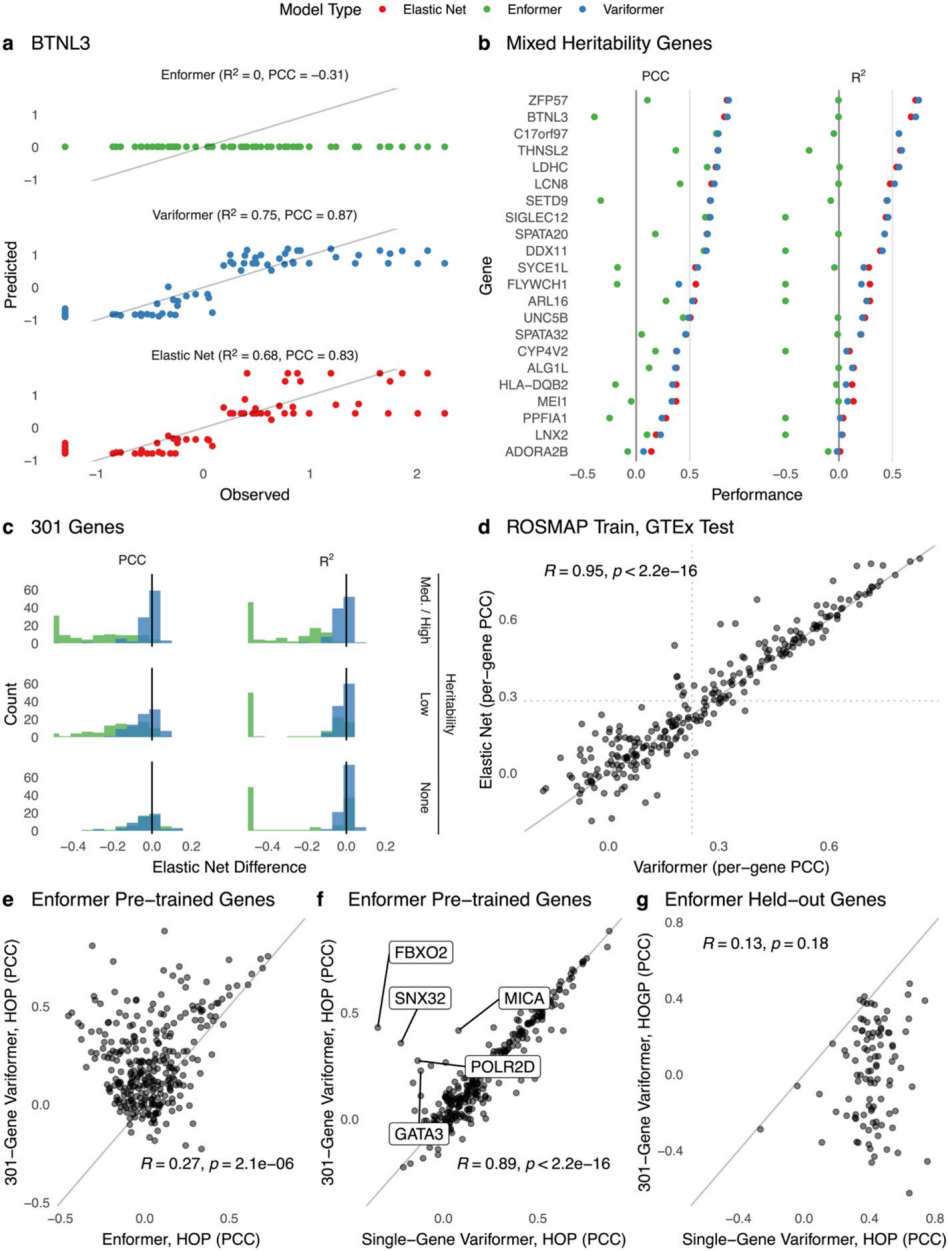
Cross-individual fine-tuning increases expression variation explained by model predictions for train genes but not unseen genes. Panels **a**-**f** display performance on held-out people (HOP) and train genes. Panel **g** displays a comparison of performance on held-out genes and people (HOGP) versus performance on HOP. **a** Coefficient of determination (R^2^) and Pearson correlation coefficient (PCC) between observed expression values and predicted expression using pre-trained Enformer, Enformer after fine-tuning on paired *BTNL3* WGS/Whole Blood RNA-seq data from GTEx (Variformer), and a penalized linear model (elastic net) fit on the same data. Each point represents an individual from the test set and their Whole Blood *BTNL3* expression, one of the 301 training genes selected for its high expression cis-heritability. Results from each model are from the same 71/670 individuals. Variformer and elastic net results come from a single model replicate to provide a clear view of the predictions. Mean performance across replicates is shown in panel (**b**). **b** R^2^ and PCC of models trained on single genes with diverse expression cis-heritabilities (as estimated by elastic nets). Variformer and elastic net models were trained on single genes and evaluated on the same genes using individuals from the test set, with performance averaged over 3 model replicates evaluated on different splits of individuals. R^2^ and PCC for Enformer are averaged over the same three test sets. Variformer and elastic net models are typically within a small margin of each other. Enformer R^2^ values are thresholded at −0.5 for axis visibility. R^2^ is expected to be lower for Enformer (Methods). **c** Enformer and Variformer performance values (PCC on left, R^2^ on right) for each of 301 training genes is subtracted from the elastic net metric evaluated on the identical test set (held-out people), and the average over 3 replicates is reported. Histograms show distributions of these deviations from elastic net performance over genes. Genes with values close to zero have similar performance to elastic nets. Genes are divided into equal sized groups based on elastic net heritability estimates (R^2^): medium/high (0.06 to 0.76; top), low (0.01 to 0.06; middle), and none (0 or less; bottom). **d** PCC of 300 genes using Variformer and elastic net models trained on single genes using paired WGS and dorsolateral prefrontal cortex RNA-seq data from ROSMAP and evaluated on the same genes and test individuals using GTEx WGS and Brain—Cortex RNA-seq data (*n* = 205). Each point represents a different gene. Performance of the Variformer and elastic net models is significantly correlated (t-test *p* < 2e-16); Variformer R^2^ is greater than elastic net for 43.4% of high heritability genes. The absolute difference between Variformer and elastic net R^2^ is less than 2% for 60.3% of high heritability genes and 62% of genes with no heritability filter. PCC values come from one model replicate. **e** PCC of Variformer jointly trained on 301 genes (y-axis) versus Enformer (x-axis), both evaluated on each of the 301 training genes. For most genes, Variformer explains more expression variability. **f** PCC of Variformer jointly trained on 301 genes (y-axis) versus Variformer trained on single genes (x-axis), both evaluated on each of the 301 training genes. Performance is very similar for multi-gene and single-gene models. A few exceptions, for which multi-gene training improved performance, are labeled. **g** PCC of Variformer jointly trained on 301 genes and evaluated on 100 held-out genes (HOGP; y-axis) versus Variformer directly trained on each of these 100 genes and evaluated on held-out people (HOP; x-axis). The 100 genes used in both evaluations have high expression cis-heritability and were not used to train Enformer or Variformer (Methods). For most genes, multi-gene fine-tuning did not achieve the same PCC on unseen genes as can be achieved by directly training on the gene

We first investigated performance on held-out people and train genes (HOP). Using Variformer, pre-trained Enformer, and elastic net models, we made blood gene expression predictions for HOP. As others have done [6, 16, 18], we evaluated model predictions for each gene in terms of the variance in expression they explain across people with the coefficient of determination (R^2^) and Pearson’s correlation coefficient (PCC). These single gene, cross-individual evaluations measure the ability of each model to differentiate between people’s expression based on their genetic variants, which are the only differences between the input DNA sequences. In agreement with prior studies [16, 18], Enformer’s predictions across GTEx individuals tend to show relatively limited variation or variation that is negatively correlated with observed values (Fig. 1a). Our fine-tuning corrected this for most genes across the range of expression heritability (Fig. 1b-c; Additional file 1: Fig. S1). Variformer’s performance metrics are consistently better than Enformer’s and generally on par with elastic nets’ (mean difference in R^2^ = 2.2% overall and 2.7% for medium and high heritability genes).

Learning curves generated by downsampling the training set indicate that this level of performance is generally achievable with 250 or fewer individuals, although the trend suggests this may improve if more individuals were available (Additional file 1: Fig. S2). This improvement is dependent on fine-tuning with personal genomes and matched expression values, as we did not see similar trends when fine-tuning Enformer with GTEx expression data while using the reference genome (Additional file 1: Fig. S3). Finally, we trained Variformer and elastic net models using paired WGS and dorsolateral prefrontal cortex RNA-seq from the ROSMAP study [25] (*n* = 742) and made HOP predictions for GTEx individuals with brain cortex RNA-seq (*n* = 205), observing good cross-cohort performance despite cortex being a less specific brain region (Fig. 1d). These results establish that deep neural networks trained on paired WGS and RNA-seq can accurately model the relationship between gene expression and cis regulatory variants after incorporating genetic variation into the training scheme.

Given these characteristics, we hypothesized that training Variformer models on multiple genes simultaneously might improve their performance. A whole blood multi-gene model based on all ~ 300 training genes outperformed Enformer on most of these genes (Fig. 1e) but was comparable to single-gene models (Fig. 1f), when evaluated on HOP. We wondered if multi-gene models might be better at generalizing to genes not used in training Enformer or Variformer, reasoning that information integrated from various loci would offer the best chance at success for this task. Although Enformer and multi-gene Variformer explained a similar amount of inter-individual gene expression variability on held-out genes and people (HOGP) to each other, their performance was still much worse than single-gene models trained directly on the same held-out genes (Fig. 1g, Additional file 1: Fig. S4). Increasing multi-gene Variformer’s training set to ~ 11,000 genes did not boost performance on HOP or HOGP (Additional file 1: Fig. S4), nor did longer 196-kb sequences (Additional file 1: Fig. S5). Likewise, using checkpointing and early stopping to monitor the ability of Variformer to predict cross-individual expression differences in unseen genes did not boost final performance on HOGP (Additional file 1: Fig. S6; Methods). Thus, the level of generalizability required to predict causal variant effects in novel loci remains an important open challenge, as this is expected of models that have comprehensively learned causal regulatory patterns.

### Fine-tuning on personal genomes improves upon previously identified model limitations

**Fig. 2:**
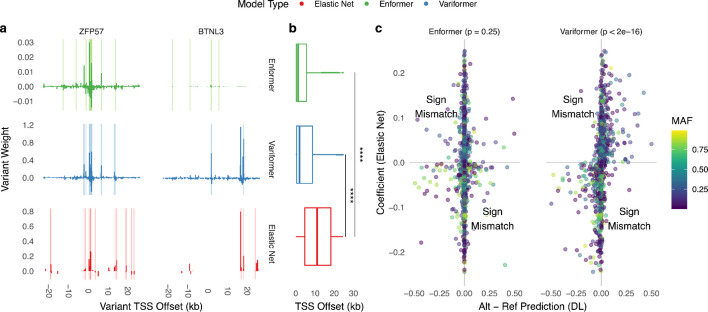
Cross-individual fine-tuning alleviates previously identified limitations of sequence-to-expression models. **a** Variant weights (elastic net: coefficient; Enformer/Variformer: alternative-reference prediction from ISM) versus variant position relative to TSS for *ZFP57* and *BTNL3*. Light vertical lines represent significant *motifbreakR*^28^ motif disruptions at HSVs. **b** Distribution of high-scoring variant (HSV) proximity to TSSs for 42/301 genes with Variformer R^2^ > 0.2. HSVs for a given gene include all SNVs with non-zero coefficients for elastic net models and the same number of SNVs with the greatest absolute ISM values for Enformer/Variformer. **c** Variant weights of elastic nets versus Enformer (left) or Variformer (right), for the same genes as in (**b**), demonstrating increased support for Variformer variant directional effects, regardless of minor allele frequency. *p*-value from Fisher’s exact test. Only SNVs whose elastic net coefficients are non-zero are shown. Enformer/elastic net and Variformer/elastic net have 60.2% and 97.4% sign agreement for weights > 0.05, respectively

Next, we investigated why Variformer achieved competitive performance on HOP. We defined High Scoring Variants (HSVs) by ranking SNVs by the absolute difference between each model’s alternate and reference allele expression predictions, then used the number of non-zero elastic net coefficients to establish a consistent HSV set size across models. While elastic net HSVs tend to be distributed over the whole window (Fig. 2a), Variformer’s and Enformer’s HSVs are generally more TSS proximal (Fig. 2a-*ZFP57;* Fig. 2b). However, Variformer’s HSV-TSS distances vary, with some genes having primarily distal HSVs (Fig. 2a-*BTNL3*). Variformer and elastic net HSVs largely have the same direction of effect, while Enformer’s scores often disagree (Fig. 2c) [16, 18], leading to negative cross-individual correlations (Additional file 1: Fig. S1). Thus, cross-individual training improved upon a key limitation of existing deep learning models.

### Variformer combines gene regulatory patterns and eQTL-like associations to predict cross-individual expression differences

**Fig. 3:**
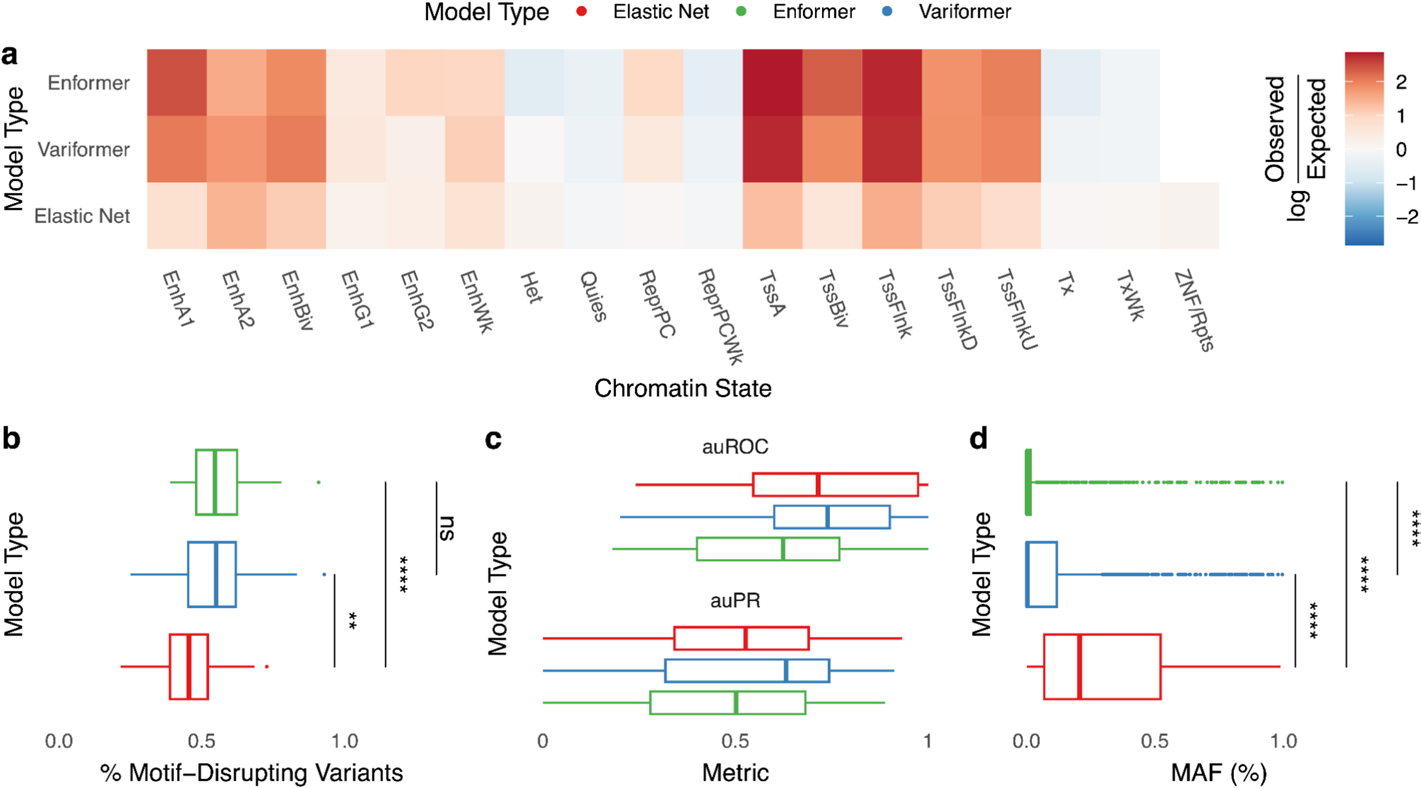
Cross-individual fine-tuning retains weighting of regulatory sequence patterns. All panels display properties of Enformer, Variformer, and elastic net HSVs for 42/301 genes with Variformer R^2^ > 0.2, evaluated on held-out people (HOP) and train genes. Significance: **** = adjusted *p*-value < 1e-4, ** < 1e-2, ns = not statistically significant. **a** Enrichment of HSVs across ChromHMM [26] chromatin states in monocyte cells from Epimap [27], averaged across 3 donors. **b** Distribution of HSVs that significantly increase or decrease the affinity of an overlapping DNA-binding protein’s motif, as measured by *motifbreakR* [28] with a p-value threshold of 5e-5. Enformer and Variformer are not statistically different, while both models identify significantly more motif-disrupting variants than elastic net. **c** Distribution of area under the Receiver Operating Characteristic (auROC) and Precision-Recall (auPR) curves for predicting CAVIAR [29] fine-mapped Whole Blood GTEx eQTLs. Absolute-valued weights for ridge regression, Enformer, and Variformer models were used alongside fine-mapped causal eQTLs to compute each metric for each gene. Variformer had the highest mean auROC and auPR (0.737, 0.53), followed by ridge regression (0.707, 0.485) and Enformer (0.611, 0.475). Ridge regression is used here instead of elastic net so that all variants are assigned a weight. **d** Statistically significant differences exist between the distribution of HSV minor allele frequencies (MAFs) of each model, with Variformer utilizing fewer common variants than elastic net but more than Enformer

We wondered if Variformer’s increased similarity to elastic nets, which form accurate predictions by memorizing the presence of important common variants without the consideration of functional relevance, would decrease Variformer’s identification of SNVs with functional evidence. Instead, Variformer’s HSVs maintain Enformer’s strong functional enrichments including active enhancer and promoter chromHMM states (Fig. 3a) and disruption of transcription factor motifs (Fig. 3b). Furthermore, Variformer’s variant weights predict fine-mapped GTEx eQTLs on par with elastic net’s and better than Enformer’s (Fig. 3c), while retaining Enformer’s ability to rank the expression of different genes (Methods, Additional file 1: Fig. S7). Thus, cross-individual, single-gene training does not notably degrade the neural network’s encoding and weighting of regulatory sequence patterns.

**Fig. 4:**
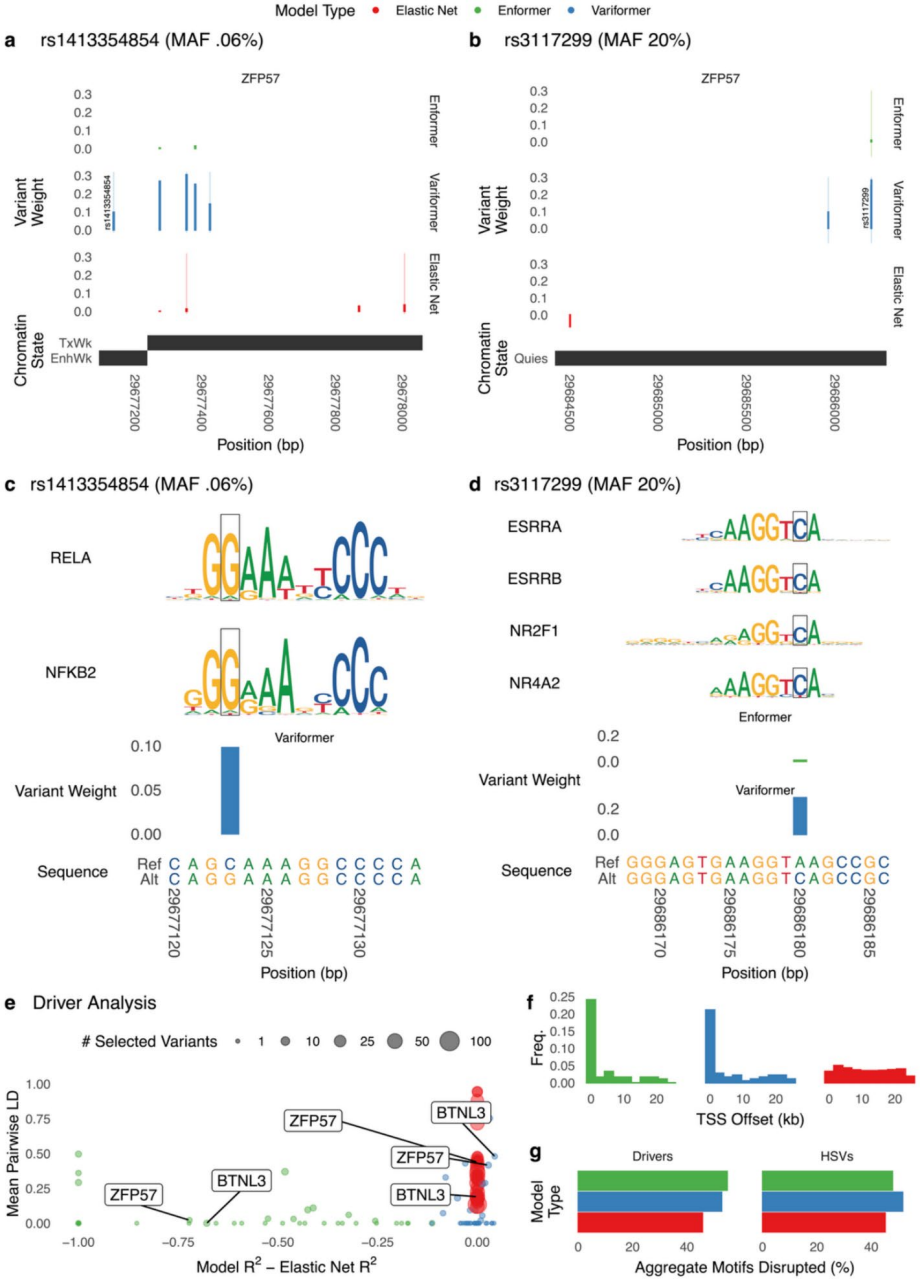
Variformer identifies HSVs that alter motifs and drive expression predictions. **a** Genomic track view of variant weights (Enformer/Variformer: ISM (Methods); elastic net: coefficient) around a rare HSV (rs1413354854; GTEx MAF = 0.06%) of *ZFP57* that falls in a ChromHMM [26] weakly active enhancer. Variformer highly weights this HSV, while Enformer and elastic net assigns moderate weights to several flanking SNVs. For each model, wide bars denote HSV weights, and thin lines represent significant *motifbreakR*^34^ motif disruptions at HSVs. **b** Genomic track view of Enformer, Variformer, and elastic net variant weights around a common HSV (rs3117299; GTEx MAF = 19.98%) of *ZFP57* that falls in a quiescent chromatin region. Variformer highly weights the HSV, while elastic net and Enformer have no HSVs nearby. For each model, wide bars denote HSV weights, and thin lines represent significant *motifbreakR*^34^ motif disruptions at HSVs. **c** Zoomed in view of rs1413354854 showing that the alternative allele creates a binding site for NFKB2 and/or RELA. Weights are shown for HSVs per model; rs1413354854 is an HSV for Variformer but not Enformer. Motif disruption identified using *motifbreakR*. **d** Zoomed in view of rs3117299 showing that the alternative allele creates a binding site for several blood transcription factors. Weights are shown for HSVs per model; rs3117299 is an HSV for Variformer and Enformer. Motif disruption identified using *motifbreakR*. **e** Model performance on HOP relative to elastic net plotted against the mean pairwise linkage disequilibrium (LD) of selected variants (Enformer/Variformer: drivers (Methods), elastic net: non-zero coefficients), with the number of selected variants indicated by dot size. For the vast majority of genes, a very small number of Variformer/Enformer driver variants are identified (resulting in a lower mean LD) compared to penalized regression with elastic nets, though forward selection could similarly be used with a non-penalized model to select a smaller number of predictive variants. Only 42/301 genes with Variformer R.^2^ > 0.2 are shown. **f** Distribution of selected variant distance from TSS (Enformer/Variformer: drivers, elastic net: non-zero coefficients). **g** Rates of motif affinity changes based on *motifbreakR* for HSVs and selected variants (Enformer/Variformer: drivers, elastic net: non-zero coefficients)

Since deep learning models tend to score rare variants higher than common eQTLs [23] and the minor allele frequencies (MAFs) of Variformer/Enformer HSVs are generally lower than those of elastic nets (Fig. 3d), we repeated the functional signature analyses after removing HSVs with MAFs < 1% or < 5% and found that Variformer’s functional enrichments persist (Additional file 1: Fig. S8). For example, the top Variformer HSVs of *ZFP57* include both rare variants (Fig. 4a,c), and common variants (Fig. 4b,d) that create stronger matches for blood transcription factor motifs. Elastic nets upweight adjacent SNVs that do not alter motifs and assign zero weight to this common variant. Next, we identified a small number of driver variants for each Variformer/Enformer model by creating a surrogate linear model with comparable performance to the full model via forward selection [16] (Fig. 4e, Additional file 1: Fig. S9). Compared to all HSVs, drivers have somewhat higher MAFs and lower pairwise LD (Additional file 1: Fig. S9). Nonetheless, they maintain the functional properties of HSVs (Fig. 4f-g). These results show that Variformer’s weights prioritize SNVs that explain expression variability across people and share many properties with the SNVs prioritized by linear models, but differ from eQTLs in the strength of their functional signatures and their broader range of allele frequencies. Thus, Variformer has useful features previously associated with both linear and deep learning models.

Next, we investigated whether Variformer utilized SNVs predominantly based on the learned association between genotype and expression – akin to elastic nets – rather than by also integrating pre-trained information about gene regulation. Instead, we found Variformer's gradient input attributions are elevated across the regions surrounding these SNVs, and include broader peaks than Enformer, supporting the possibility of motif learning after fine-tuning (Additional file 1: Fig. S10). In addition, Enformer’s attributions around driver SNVs are more similar to control SNVs (Additional file 1: Fig. S10), consistent with the idea that Enformer’s poor accuracy and low variability of inter-individual expression predictions are in part due to weak attributions around driver SNVs [16, 18]. We also observed decreased cross-individual prediction accuracy when training Variformer from random initial weights (Additional file 1: Fig. S11), and almost no overlap with HSVs from the fine-tuned model (8% Jaccard similarity). Together with our other results (Figs. 1a, 3 & 4, Additional file 1: Fig. S8), these findings are consistent with the notion that Variformer prioritizes SNVs based on both presence in gene regulatory elements (due to Enformer’s pre-trained weights) and association with expression (due to fine-tuning).

### Results are robust against alternative modeling decisions and architectures

We next investigated whether different modeling decisions would change our findings. We first modified our loss function, which includes both cross-individual and mean-squared difference terms (Methods), to use either one or the other. We found that the former helped expedite convergence, the latter incentivized proper prediction magnitudes, and including both terms in the loss helped Variformer achieve a combination of these properties (Additional file 1: Fig. S12). We also modified the learning rate (Additional file 1: Fig. S13), as well as the effective batch size via gradient accumulation (Additional file 1: Fig. S14), finding that these changes led to faster convergence but minimally impacted final HOP performance. Finally, we trained Variformer while keeping Enformer’s original parameters frozen, observing slower convergence and poorer overall HOP performance (Additional file 1: Fig. S15). None of these modifications led to an improvement in HOGP accuracy.

**Fig. 5:**
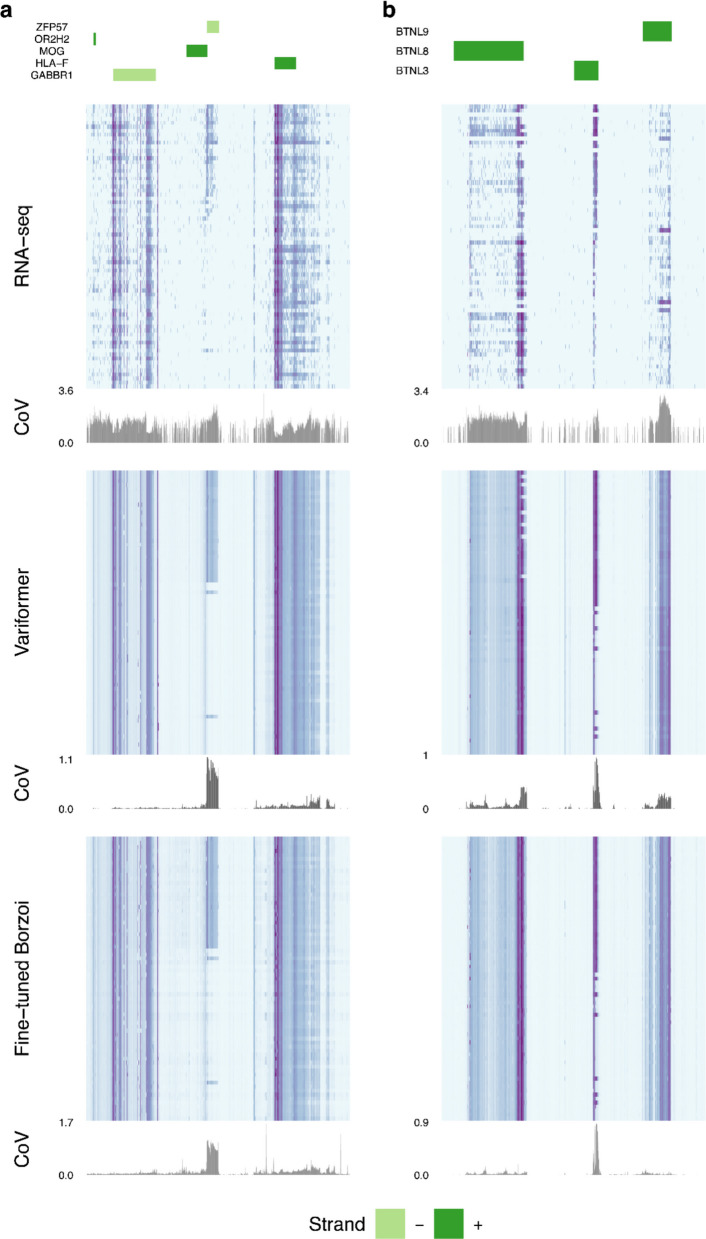
Fine-tuning with alternative architectures. **a** Genomic region centered on the *ZFP57* gene showing flanking genes (top), observed RNA-seq coverage for HOP (second from top; purple: high coverage, light blue: low coverage) with its coefficient of variation (CoV), predicted coverage on the same HOP using fine-tuned Enformer (Variformer; middle heat map, same color scale) and its CoV, and predicted coverage using fine-tuned Borzoi (U-net architecture; bottom heat map, same color scale) and its CoV. Rows of heat maps correspond to different held-out individuals, sorted in all three heat maps from highest to lowest expression at the *ZFP57* TSS. Both models predict de-noised versions of the observed RNA-seq signal while generally capturing predictive, high CoV regions across people. Fine-tuned Borzoi captures a high CoV region downstream from the ZFP57 TSS that Variformer misses. **b** Genome region centered on the *BTNL3* gene showing the same analyses as in (**a**). Variformer captures expression variance at *BTNL8* and *BTNL9* that is missed by fine-tuned Borzoi. Both models capture high CoV regions at *BTNL3* and a region containing the HSV described in (Fig. 2A)

Next, we expanded our fine-tuning strategy to the Borzoi model [30] which has a different architecture from Enformer, was pre-trained directly on RNA-seq rather than CAGE-seq, and accepts longer sequences (524 vs 196 kb) while outputting higher resolution tracks (32 vs 128 bp). Borzoi models fine-tuned on GTEx blood RNA-seq (scalar values) achieve comparable HOP performance to Variformer and elastic nets (Additional file 1: Fig. S16). We also fine-tuned Enformer and Borzoi on blood RNA-seq read coverage tracks. Both models capture observed coverage variability at gene promoters and ignore it at intergenic regions, but they sometimes under-estimate inter-individual expression differences at different locations (Fig. 5). Thus, both architectures perform well at de-noising but may mistake technical and biological variation under different circumstances. This suggests that further minor improvements to sequence length, output resolution, expression data encoding, and architecture may not offer significant performance gains over linear models for cross-individual expression prediction.

## Discussion

We showed that training on personalized genome data and paired expression from hundreds of individuals dramatically improves deep learning models’ ability to predict expression differences amongst unseen individuals, in agreement with other studies [31, 32]. Furthermore, Variformer prioritizes genetic variants across the allele frequency spectrum that disrupt motifs, fall in annotated regulatory regions, and have functional evidence for modulating gene expression. In terms of causal variant prediction, Variformer retains Enformer’s utilization of SNVs in candidate regulatory elements, while gaining linear models’ ability to correctly predict the direction of SNV effects on gene expression and to prioritize fine-mapped eQTLs. Additionally, Variformer HSVs include both rare and common variants. These characteristics suggest that Variformer variant weights hold promise for causal variant detection, though future high-throughput experimental validations are needed to test this hypothesis.

The Variformer approach has several important limitations. First, similar to eQTL models, Variformer only estimates the genetic component of gene expression variability and performs best on genes with high cis-heritabililty. Second, we only investigated SNVs in this study; it will be important to extend Variformer to insertions, deletions, and inversions, because they are major contributors to expression variability [33]. Third, Variformer is competitive with existing models at complementary tasks (e.g., using common variants to impute expression of unseen individuals for elastic nets, functional variant prioritization across the MAF spectrum for Enformer), bridging the gap between these modeling approaches. However, it does not out-perform either method and hence has room for improvement. Furthermore, fine-tuning did not address the ambitious task of predicting the expression variability of unseen genes (which no model currently does), even when trained jointly with thousands of genes or larger input sequences. This lack of generalizability may be due to Variformer upweighting changes in motifs that have limited or even opposing effects on other genes’ expression, or because of insufficient data. More specifically, we observed an abundance of genes where both elastic net and Variformer models struggled to learn inter-individual differences (Fig. 1c). The prevalence of these genes may explain why training on more genes did not improve HOGP performance; their low cis-heritability offers little information for generalizing to unseen genes. Thus, while we trained jointly on as many as ~ 11,000 genes, the effective number of learnable genes is likely below the number necessary to improve accuracy on HOGP and that HOGP generalization may require different modeling strategies. A possible explanation for the similar performance of elastic nets and Variformer on HOP is that they may be near the theoretical maximum for these genes given the sample sizes in our test set, our not modeling insertions and deletions, and the allele frequencies of SNVs that explain expression cis-heritability. It should be noted that the best-in-class performance on HOP, by both Variformer and elastic net models, can often be attributed to the identification of important common variants (e.g., eQTLs) by these models in unseen individuals that were also seen during training. While elastic net models explicitly rely on only these associations between genotype and expression, we report that Variformer also prioritizes SNVs by integrating pre-trained information about gene regulation. Thus, feature leakage is a concern for both modeling classes, but Variformer’s prioritized SNVs have some additional evidence of functionality.

Several recent pre-prints support our conclusions. Rastogi & Reddy et al. [31] and Spiro & Tu et al. [32] also trained deep sequence-to-expression models using paired whole genomes and gene expression measurements. The former fine-tuned Enformer with a different loss function than we used, and the latter trained a simpler CNN-only architecture from scratch. Another recently developed model, CLIPNET, was trained on paired personal genomes and transcription initiation data, and the authors found this scheme improved unseen variant effect predictions [24, 34]. This approach relies on predicting the local effects of regulatory syntax onto transcription initiation from short sequences. Predicting steady-state RNA, however, requires the model to consider longer range interactions between various regulatory elements and a large set of genetic variants, which has proven to be challenging for current models [17]. The consistency of performance results across these studies indicates that the findings we report in this manuscript are robust to a wide range of modeling decisions.

Our results suggest several future directions that may help sequence-to-expression models realize their full potential to decode gene regulatory logic. For example, performance may be bolstered by combining cohorts like GTEx with massively parallel reporter assay (MPRAs) and high-throughput genome editing perturbation data which includes many rare and de novo variants. Indeed, others have suggested the variation present in the human genome is insufficient for learning causal patterns from sequence [35]; larger libraries of synthetic variants may help bridge this gap, though addressing assay biases relative to endogenous variants would be essential. New architectures and loss functions optimized to distinguish technical and biological variability are also worth exploring, as are approaches to better capturing the effects of distal variants.

## Conclusions

Together, our findings demonstrate that incorporating genetic variation via fine-tuning corrects previously documented issues for cross-individual expression prediction, identifying functionally enriched variants with predictive power comparable to the limits of heritability established by penalized linear models. We show that this scheme does not lead to improved predictions on held-out people and genes, highlighting a gap for future research to resolve.

## Methods

### Inputs

For each individual in ROSMAP and GTEx, we used *bcftools consensus* [36] to replace reference genome (hg38) nucleotides with single-nucleotide variants (SNVs) present in their unphased VCF file. All SNVs were used, without allele frequency filtering, to standardize the genetic variation seen by each approach. This led to two consensus sequences per individual; one contains alternate alleles only for homozygous SNVs and reference alleles elsewhere, the other contains alternate alleles for heterozygous SNVs and homozygous SNVs. If phased haplotypes were available, Variformer could work with those.

During training and evaluation, we used 49,152 basepair (bp) input sequences that are 4 × shorter than Enformer is equipped to handle (196,608 bp) to enable faster and larger batches within the available GPU memory. For each gene, we fetched an individual’s two 49-kilobase (kb) consensus sequences centered on the gene’s TSS (GENCODE [37] v26). We one-hot encoded each sequence, and used the average of two one-hot encoded matrices as our input [24]. While other approaches are possible [7], we found this representation to be reasonable because: (1) it allows for one input per person, speeding up training and evaluation; (2) since the variants are unphased and not matched to allele-specific expression, it avoids the situation where the model is trained or evaluated on two different sequences from the same person being paired with the same gene expression target; (3) the resulting encoding represents whether a person has no dosage (0/0; 0), half dosage (0/1; 0.5), or full dosage (1/1; 1) of any particular SNV.

### Targets

For GTEx “Whole Blood” and “Brain—Cortex” expression, we downloaded tissue normalized data from GTEx [12] v8. ROSMAP TPM values were normalized using the same procedure (https://gtexportal.org/home/methods; https://github.com/broadinstitute/gtex-pipeline/). Genes with less than 0.1 TPM or fewer than 6 reads in 20% of samples are removed; their trimmed mean expression is computed and an inverse normal transform is applied. Each gene’s mean expression value is near 0 and represents a deviation away from the mean in either direction. During training and evaluation, each individual’s tissue-specific gene expression value is represented as a scalar target value.

### Fine-tuned Enformer architecture and pre-trained weights

We fine-tuned the pre-trained pytorch implementation of Enformer (https://github.com/lucidrains/enformer-pytorch; v0.8.8), using the provided HeadAdapterWrapper class, with num_tracks set set to 1, post_transformer_embed set to False, and the output activation set to the identity function. This bypasses the original human and mouse Enformer heads, passing instead through a linear layer with no activation. When using 49,152 bp DNA sequences, Enformer’s embeddings are 384 (number of bins) × 3072 (number of features). After passing through the linear layer, the output has the shape 384 × 1. We converted this sequence into a scalar by keeping one of the TSS-overlapping center bins (bin 192), and using the value at this position for the final prediction. This value was therefore used to compute the loss during training, and was compared against observed expression values during evaluation. Although we use this scalar value as the final prediction, we note that information from regulatory elements at other positions are also mixed into this position’s embedding via attention. Rastogi & Reddy et al. [31] used attention pooling to achieve a weighted linear combination over the ten central bins and achieved results that were mostly consistent with ours, suggesting this decision was not critical for our results.

### Definition of TSS-centered genomic intervals for training and evaluation

We retrieved the genomic intervals corresponding to training, tuning, and testing for Enformer [15]. First, we downloaded the Basenji2 [38] genomic intervals from https://console.cloud.google.com/storage/browser/basenji_barnyard/data. Next, we extended these 131,072 bp intervals to 196,608 bp. Although this was not done in Avsec et al., we additionally dropped genomic intervals from the train set if, after extending the sequence length, any of these intervals leaked into the tuning set or test set. Likewise, we dropped intervals from the tuning set if extending them caused leakage into the test set.

Of these remaining genomic intervals, we next found 196,608-bp regions that are centered on the TSS of a protein-coding gene and are completely contained within either the train, tune, or test set. We used GENCODE [37] v26 annotations to define TSS coordinates of protein-coding genes. We omitted from consideration 228 train, 21 tune, and 36 test genes that were within ± 64 bp of another gene’s annotated TSS. We made this decision because DNA sequences from these genes’ genomic intervals would include the TSS for multiple genes within the same Enformer output bin (each bin along the sequence axis of Enformer’s output represents 128 bp of original sequence). We kept only genes with sufficient expression (see Targets), leaving us with 11,429 train, 1057 tune, and 1398 test genes for models to be trained on GTEx Whole blood and 12,292 train, 1194, tune and 1492 test genes for models to be trained on ROSMAP DLPFC. When training or evaluating 49,152-bp sequences, we trimmed the left and right edges of these 196,608 bp regions to keep only the center portion of the sequences containing the annotated TSS.

### Selection of train and test genes

To select train genes, we first fit elastic nets (separately for ROSMAP DLPFC and GTEx Whole Blood) on approximately 1000 genes in Enformer’s original train set, and computed R^2^ using one cross-validation fold (see Training Variformer Models and Fitting Penalized Linear Models). We then split the resulting R^2^ distribution into 10 equally sized bins and attempted to select 30 genes from each bin, starting with the largest R^2^ bin. If there were less than 30 genes available, more would be sampled from the next bin to compensate. For each of ROSMAP and GTEx Whole Blood, this left us with 300 genes whose elastic net R^2^ approximate the underlying distribution of genes in that tissue, while keeping as many high heritability genes as possible given training time. We added an additional gene, *PPIF*, to the whole blood train set, because of the availability of published perturbation data around this locus measured in THP-1 cells [39], to enable future experiments that benchmark variant weights from different models against published perturbation data at this locus. In total, this generated 301 genes for training models using GTEx Whole Blood data and 300 genes for training models using ROSMAP DLPFC data.

To select test genes, we similarly fit elastic nets on every gene in Enformer’s validation (i.e., tuning) and test set, and selected up to 100 genes whose elastic net R^2^ was at least 10%. This led to 82 test genes in ROSMAP DLPFC and 100 in GTEx Whole Blood. We prioritized test genes with strong elastic net performance so we would be able to confidently detect accurate predictions (measured by PCC or R^2^) if they occurred. These are the held-out genes in Figs. 5 and S5. For our experiment involving training on 11,429 train genes, we used all genes in the train set (see Definition of TSS-centered genomic intervals for training and evaluation).

### Training variformer models

We fine-tuned Enformer with a learning rate of 5e-6, bf16-mixed precision, and clipping the gradient’s global norm to less than 0.05 using Pytorch Lightning (https://lightning.ai/docs/pytorch/stable). We fine-tuned with 49,152-bp sequences and a batch size of 32 on an H100 GPU. We used gradient accumulation for 4 batches to achieve an effective batch size of 128. All samples in each batch corresponded to data from different people but the same gene, and the same gene was used for all batches until the 4 iterations of gradient accumulation had completed. We chose to include only different people but the same gene in each batch because our loss function emphasized small expression differences between people (see Loss Function) to help learn the subtle impact of genetic variants on expression.

An epoch was defined as a step through all genes in the train set and the maximum number of unique individuals that fit into an integer multiple of the effective batch size of 128, which was 512 individuals for both the GTEx Whole Blood and ROSMAP training sets. For single gene Variformer models, an epoch would end once these individuals were stepped through, and the tuning epoch would then proceed.

We performed threefold cross validation by splitting the total number of individuals with matched WGS and RNA-seq data into train/tune/test splits. For models trained on GTEx Whole Blood data, this was an 80/10/10% split of 670 individuals. Models trained on ROSMAP DLPFC data were trained using a 90/10% train/tune split of 742 individuals and then tested on 205 unseen individuals for which there was WGS and GTEx Brain—Cortex RNA-seq data.

We evaluated the average R^2^ on all genes in the train set and individuals from the tuning set every epoch, and implemented early stopping if this value did not increase with a patience of 20 epochs. After training, the model checkpoint with the greatest average R^2^ on the tuning set, among genes in the train set, was used for evaluation with the test set and subsequent experiments. The test set included test set individuals (described above) and test set and/or train set genes, depending on the experimental goal.

To train jointly on 11,429 genes, we used the “ddp_find_unused_parameters_true” strategy in Pytorch Lightning to train with 8 H100 GPUs. To support our loss function, which emphasizes differences between people for a given gene (see below), we implemented a custom sampler to divide these genes equally among the 8 GPUs and ensure that a batch received by any device always includes data from different individuals but for the same gene. We used a batch size of 16, and evaluated the model on all train genes and individuals from the tuning set every 2 epochs. We stopped training after 33 epochs (7 days), when PCC evaluated on train genes and tuning individuals stopped increasing. We trained only one model due to the computational resources required.

Our “tuning” set is equivalent to what is commonly referred to as a “validation” set. We choose to use the word “tuning” to avoid confusion between the meaning of “validation” in the contexts of machine learning and biological experiments. In the former, it is an intermediate dataset used to estimate performance during training and avoid over-fitting. In the latter, it is often referred to as the final experimental group used to confirm results. Here, the tuning set is used to estimate performance during training, while the test set is the name of the final set of held-out samples used to evaluate trained models.

### Loss function

Our loss function contained two components. First, the mean-squared error between observed and predicted expression values. Second, we took the pairwise difference between each unique pair of observed expression values within the batch, and performed the same operation for predicted expression values. We then took the mean squared difference between these two pairwise difference vectors. Minimizing the first component incentivizes predictions to be similar to observed values. Minimizing the second component incentivizes the predicted difference in expression between different people to be similar to their true differences. Because all samples in a batch corresponded to different people but the same gene, we reasoned this would emphasize differences conferred by genetic variants onto gene expression, as opposed to differences in expression between different genes. Finally, we took the weighted sum of the two components, weighting each component by one half.

### Calculation of Pearson correlation coefficient and R^2^ across people

We applied the same three cross-validation splits (see Training Variformer Models) to elastic net and Variformer models, thereby maintaining consistency between the individuals used to train and evaluate them for different model replicates. For example, Variformer and elastic net models based on the first cross-validation split used identical train/tune/test individuals, and likewise for replicates based on the other two splits. We evaluated Enformer on the same test sets. Unless otherwise specified, for all three model types, we report Pearson correlation coefficients (PCCs) and R^2^ (coefficient of determination) obtained from the average of these three test sets for when evaluating on GTEx Whole Blood. When evaluating on GTEx Brain—Cortex, model replicates were not trained to conserve computational resources. Thus, one model per gene is trained on ROSMAP and evaluated on 205 individuals with GTEx Brain—Cortex WGS & RNA-seq data. We report Enformer’s performance on the same 205 individuals. Unless otherwise specified, we used 49 kb genomic windows to train and evaluate elastic net models and Variformer models, and evaluate Enformer models, to facilitate further consistency. We either evaluate models on held-out people and the genes they were trained on (HOP) or, for Variformer and Enformer, we also evaluate on held-out people and genes (HOGP). Unless otherwise specified, analyses were conducted on HOP.

The PCC and R^2^ across people were calculated separately for each gene through the following procedure. For a given gene, we created a vector of observed gene expression values for all individuals in the test set as well as a second vector of predicted values for the same individuals, in the same order. For the fine-tuned Enformer models (Variformer), the predicted values were generated by passing in each person’s DNA sequence for that gene, and the scalar output was used (see Fine-tuned Enformer architecture and pre-trained weights for more details on obtaining a scalar output). For Enformer, we compared the ‘CAGE:blood, adult, pool1’ (track 4950) output to observed GTEx Whole Blood expression values, and the 'CAGE:brain, adult' (track 4980) output to observed GTEx Brain—Cortex expression values, as others have done [16]. We took the sum over the three center bins of the output, and this value was added to the second vector of predicted values. For 49,152-bp input sequences this was bins 191, 192, and 193 out of 384. We note that the R^2^ for Enformer is expected to be lower, as this model was trained to predict count values rather than normalized TPM values; Enformer will be disadvantaged with R^2^ even if it ranks individuals’ expression values correctly, which will be captured by PCC. Finally, for elastic nets, the predicted values were obtained by passing in each test individual’s genotype matrix to a model fit on the same gene, including only SNPs within the 49,152 bp region centered on that gene’s TSS. Using these vectors of observed and predicted expression values, we computed correlation using *scipy.stats.pearsonr* and R^2^ using *sklearn.metrics.r2_score*. We note that PCC is not the square root of R^2^ for non-linear models, motivating us to report both performance statistics.

### Driver analysis and in silico mutagenesis

For any given gene, we performed in silico mutagenesis (ISM) using fine-tuned models and base Enformer by first performing one prediction with the TSS-centered reference genome sequence (49,152 bp). Then, for each observed SNV within the input window in any individual in GTEx, we took the same sequence and replaced the reference nucleotide with the alternate allele nucleotide at the same position, and performed another prediction. For Enformer, we kept predictions from the 3 central bin positions (bins 191, 192, and 193), performed a sum over these bins, and the resulting value was treated as the final prediction. For Variformer models, because they were optimized to only use the center bin position (bin 192), we kept only this value. For each SNV, the ISM score (variant weight) was defined as the predicted value from the sequence with the alternate allele minus the one from the reference sequence. Absolute scores were used in analyses that rank SNVs, while the signed ones were used for comparison to elastic net coefficients.

To identify driver SNVs, defined as those that linearly approximate the model’s predictions for a given gene in a desired group of people, we followed a similar forward selection procedure to that in Sasse et al. [16] To identify drivers, we used only individuals from the test set (i.e., the held-out set of ~ 70 people for Whole Blood GTEx models or the 205 individuals with WGS and GTEx Brain—Cortex RNA-seq data for ROMSAP models), to identify variants that explain R^2^ and PCC in these individuals. We deviated slightly from the Sasse et al. procedure by including driver SNVs, as opposed to iteratively including all SNVs, to the linear approximation while searching for drivers.

### Fitting penalized linear models

Linear models were fit using a scikit-learn [40] pipeline consisting of a VarianceThreshold transform followed by an ElasticNetCV or LassoCV model with max_iter increased to 2000. Models were fit on GTEx or ROSMAP BCF files encoded as a genotype matrix with homozygous reference = 0, heterozygous = 1, and homozygous alternate = 2. We fit and evaluated these models using the same cross-validation folds as Variformer.

### Selection of HSVs

To improve variant comparisons across model types, the number of non-zero linear model variant coefficients per gene was computed. An equal number of variants per gene was selected for Variformer and Enformer using the highest scoring variants defined by the absolute value of the alternate—reference allele prediction. Analyses using these high scoring variants are thus comparing an equal number of variants per gene across model types. Variformer and elastic net HSVs come from those trained on the first cross-validation split.

### Borzoi analysis

Transfer learning was used to train single-gene models to predict scalar normalized expression values across people utilizing a Borzoi [30] trunk initialized with weights from model0_best.h5 (fold0 only). Embeddings were fed into a linear output layer trained with learning rate 1e-3 and early stopping. Separately, fine-tuning was used to train single-gene Borzoi models to predict expression tracks (rather than single values) across people. Output layers were replaced with a dense layer having softplus activation. The Borzoi trunk was initially frozen for 5 epochs to update the new output layer weights, then all layers were made trainable for 20 epochs with early stopping.

### *MotifbreakR* analysis

Variant-induced changes to the binding affinity of TF motifs was calculated using *motifbreakR* [28] with HOCOMOCO [41] v11 and a *p*-value threshold of 5e-5.

### ChromHMM analysis

Enrichment of HSVs for epigenetic states was computed using ChromHMM [26] annotations from Epimap [27], averaged over 3 peripheral blood mononuclear cell samples. HSVs for each model were overlapped with state annotations, creating an observed number of overlaps per gene/state. An expected number of overlaps per gene/state was computed by first dividing the total length of a state’s annotations that overlap the 49 kb gene window by the total length of all annotations overlapping the window. This ratio was then multiplied by the number of HSVs per gene. Enrichment per model/state was defined as the log of the observed over the expected number of states.

### auROC/auPR analysis

For high heritability genes, CAVIAR [29] high-confidence fine-mapped whole blood eQTLs (FM-eQTLs) from GTEX were used as positives. For each positive, 5 distance-matched non-FM eQTLs were used as negatives. For each model type and gene, we computed the area under the Receiver Operating Characteristic (auROC) and Precision-Recall (auPR) curves using the FM-eQTL annotation and the absolute value of the model’s variant weight.

### Training with downsampled donors

For each cross-validation fold (see Training Variformer Models), we trained Variformer models using only the 25%, 50%, 75%, or 100% of the training data, with each larger group inclusive of the previous smaller group. Individuals used for tuning and testing were not downsampled. Three model replicates were trained for each gene and downsampled group, and the mean of PCC and R^2^ over the replicates was computed. We used 40 genes for this analysis, selecting 20 genes with the highest elastic net R^2^ (averaged over three model replicates) from our initial set of 301 Whole Blood training genes (see, Selection of train and test genes) and 20 other random genes from the same set. Variformer models were trained using GTEx Whole Blood data.

### Predicting average expression of test genes

We first trained 25 single-gene Variformer models and one 301-Gene Variformer model using 196,608 bp personal genome sequences and Log_2_(TPM + 2) values. Due to the longer input sequences, we used a batch size of 4, which is smaller than the batch size of 32 we used when training with 49,152 bp sequences. The 25 genes used to train each single-gene Variformer model were selected randomly from the train set. All other aspects of training were the same as described in the Training section.

We retrained Variformer models on these TPM values because Variformer was previously trained on expression values that were transformed to appear normally distributed with a mean of 0 (see Targets) in order to emphasize within-gene differences between people. Thus, the inherited knowledge of baseline gene expression from Enformer may have been compromised after fine-tuning, disadvantaging Variformer on the task of predicting average expression of different genes. Conversely, Enformer is disadvantaged by having not been trained on personal genomes.

To evaluate the ability of Variformer to predict the average expression of unseen genes, we first calculated the mean Whole Blood log_2_(TPM + 2) value, among all 670 GTEx individuals with WGS and Whole Blood RNA-seq data, for each of the 1858 genes in the test set. We note that this test set is larger than the set of 1398 genes from which we sampled test genes for evaluation on HOGP (see Definition of TSS-centered genomic intervals for training and evaluation) because it includes additional low-expressed genes in order to more closely match the set of genes used to evaluate Enformer. Then, for each of these genes, we passed the 196,608-bp hg38 reference genome TSS-centered sequence and formed a prediction with Enformer, and each of the Variformer models. Similar to predicting Whole Blood expression from personal genome sequences, we used the ‘CAGE:blood, adult, pool1’ (track 4950) Enformer output and summed over the three TSS-centered bins (191–193). This left us with one observed and predicted average gene expression value for each gene and model. We then took the correlation between the predicted and observed average expression value across the 1858 genes for each model.

### Training Variformer models from randomly initialized Enformer weights

We trained one 301-Gene Variformer model and five single gene Variformer models with random starting weights. The five selected genes were selected on the basis of having among the greatest R^2^ achieved by elastic net models. The architecture and training procedure of these models was the same as was used for other Variformer models reported in the main figures of this manuscript (see Training Variformer Models, Loss Function, and Fine-tuned Enformer architecture and pre-trained weights), except these were trained using 196,608 bp sequences instead of 49,152 bp sequences. To train from random weights, we instantiated an `Enformer` class object from enformer-pytorch, rather than using the `from_pretrained` function.

We also attempted different hyperparameters, reasoning that a change may be necessary to improve performance when training from random initial weights. Therefore, we shortened the input sequence to 49,152 bp, which led to faster training and iteration. We also disabled early stopping to accommodate for the possibility that substantially more weight updates are necessary when training Enformer from random initial weights. Finally, we used a greater learning rate (5e^−04^). These hyperparameters were used for Additional file 1: Fig. S11 D-F.

### Training Variformer models using the reference genome and average GTEx expression values

We trained a 301-Gene Variformer by passing in the 49,152 bp reference genome (hg38) TSS-centered sequence during training. Target gene expression values were log_2_(TPM + 2) values, averaged among individuals in the train set.

While we attempted to match the training procedure as closely as possible to the personal genome training setup, some changes were necessary. Specifically, we made the following changes:

1. Our loss function, which includes a cross-individual component, is incompatible with training on only the reference genome. We altered the loss to only include the mean-squared error between observed and predicted values.
2. We trained on log_2_(TPM + 2) target values, because our original target values are transformed to be centered around 0 and emphasize within-gene differences between people. We implemented this change to avoid the situation where the target value for each gene was around 0, which would not be conducive to training.
3. We passed in the reference genome and average expression (calculated among individuals from the tuning set) during the tuning phase. Since personal genomes were not being used, we could not monitor the R^2^ or PCC between predicted and observed values of individuals in the tuning set, which was done to train Variformer models in the main text. Instead, we monitored the loss from this tuning set. While we also attempted to monitor predictions from unseen genes from the tuning set, we did not observe any difference. We report performance from a model trained while monitoring performance on train genes, to more closely mirror our setup using personal genomes.
4. We implemented early stopping with a patience of 20 epochs and a minimum improvement of 0.01.

### Testing various architectural and training decisions

We attempted many variations of our training procedure, to assess the importance of our training and architectural decisions.

### Choice of genes to monitor for early stopping and checkpointing

Throughout the rest of this manuscript, we monitored the average R^2^ between predicted and observed expression among genes in the train set and individuals in the tuning set. When this value stopped improving, training was stopped and the best checkpoint was used for evaluations on the test set (evaluations on HOP and/or HOGP; see Training Variformer Models).

In Additional file 1: Fig. S6, we also attempted to monitor 100 genes from the tuning set (genes fully contained within Enformer’s original validation set), to assess whether the resulting model would generalize better to unseen genes in the test set during evaluations on HOGP. We did this by monitoring the average PCC between predicted and observed expression among these 100 tuning set genes in individuals in the tuning set. These genes were selected by taking genes within Enformer’s original validation set, evaluating the R^2^ performance an elastic net model achieves in predicting their expression in unseen people, and selecting the top 100. We chose to evaluate PCC rather than R^2^ on these unseen genes because PCC is more sensitive to whether the model can predict the correct trend in expression values across people, while R^2^ is additionally sensitive to the scale of the predictions. We noticed Variformer struggle to form predictions with proper scale for unseen genes, so we opted to monitor the more forgiving PCC metric, which would still increase if Variformer correctly predicts gene expression differences in HOGP.

### Loss function modifications

Our loss function includes both a mean-squared difference component and a cross-individual component (see Loss Function) that are equally weighted. In Additional file 1: Fig. S12, we assessed the importance of each of these components by using only one or the other while training a 301-Gene Variformer model. We left all other aspects of training the same.

### Learning rate

In Additional file 1: Fig. S13, we evaluated 301-Gene Variformer models after training with four different learning rates: 5e-4, 5e-5, 5e-6 (used in the rest of this manuscript), and 5e-7. We only report results from the latter three experiments, because the 5e^−4^ learning rate model converged to predict constant expression values for most input sequences, which impacted axis visibility.

### Gradient accumulation

Throughout the rest of this manuscript, we trained with a batch size of 32 and accumulated gradients over four batches to achieve an effective batch size of 128. In Additional file 1: Fig. S14, we also attempted accumulating gradients over two batches to achieve an effective batch size of 64, as well as over one batch (i.e., no gradient accumulation).To perform this gradient accumulation, we used the ‘accumulate_grad_batches` attribute of pytorch lightning Trainer class, setting this value to four, two, or one, respectively.

### Freezing enformer’s trunk weights

Throughout the rest of this manuscript, we trained Variformer models by fine-tuning Enformer’s trunk weights (i.e., all of Enformer’s weights except for the output heads, which we replaced). In Additional file 1: Fig. S15, we also attempted keeping these weights frozen, and only updating the weights of a newly instantiated linear layer (see Fine-tuned Enformer architecture and pre-trained weights). To freeze Enformer’s trunk weights, we utilized the built-in support for this objective within the enformer-pytorch HeadAdapterWrapper class, by setting ‘freeze_enformer = True’ within the forward method of the class object.

### Input sequence attribution (Input gradients)

As the goal of this analysis was to evaluate whether Variformer achieves improved accuracy on HOP by relying on simple QTL-like associations (rather than the motif any variant resides within), we focused this analysis on genes where performance was high, (R^2^ > 0.2 averaged over single-gene Variformer model replicates), and there was therefore a greater potential for overfitting on QTL-like associations. From this pool of genes, we selected 15 genes at random for this analysis. Using the 49,152 bp TSS-centered region around each of these genes, we computed the gradient with respect to the input DNA sequence. We standardized these gradient attributions to ensure comparability between gradient attributions coming from different genes, whose gradient magnitudes may differ due to differences in gene expression variability. We conducted this analysis using three 301-gene Variformer replicates and Enformer. For each model and each gene, we computed the gradient with respect to the input DNA sequence through the following procedure:

1. Respecting the fact that Enformer was trained using reference genomes, while Variformer was fine-tuned using personal genomes, we computed the gradient x input using the the human reference genome (hg38) as well as with personal genomes from the test set for all models. When using personal genomes, we took the mean over the personal genome axis. This led to two input gradient matrices (one from the reference background and another from personal genome backgrounds), each of shape (49,152 × 4). These matrices were transformed independently in steps 2 & 3.
2. We then took the absolute value then the maximum value along the nucleotide axis. This amounts to converting the 4 possible nucleotides at each position into a maximum attribution magnitude to summarize the importance of that position. The resulting vector is 1-dimensional and length 49,152.
3. We applied a z-score transformation to this vector, referring to this transformed vector as the “Standardized Attribution”. The value at each position along this vector intuitively represents that position’s importance relative to the average taken from all positions.

We used driver SNPs and HSVs that were assigned to each model. Control SNPs were found for each driver and HSV by starting with the initial pool of SNPs observed in our GTEx cohort. From that initial pool, we sampled a variable number of control SNPs for each driver/HSV using the following criteria:

1. They must be within the 49,152 bp window around the same gene as the matched driver/HSV.
2. They must be within ± 2.5 kb of the driver/HSV SNP’s distance from the TSS.
3. Their minor allele frequency must be within 10% for drivers, or 0.5% for HSVs. The difference in stringency between driver controls and HSV controls respects the expectation that large effect size HSVs are typically rarer than driver SNPs (Additional file 1: Fig. S9c,d), which must be common by definition. We were therefore more lenient with the minor allele frequency for driver controls to avoid sampling an insufficient number of controls.

Note that each Variformer model replicate may have been assigned slightly different driver SNPs or HSVs. Since there are no additional Enformer model replicates, there is only one set of HSVs assigned to Enformer. But since we used individuals from the test set to define drivers (see Driver Analysis and in silico Mutagenesis), and there were three different test sets (identical to the three Variformer model replicate test sets), Enformer was assigned three sets of driver SNPs, using personal genomes from each of the three different test sets. Likewise, we calculated personal genome gradients for Enformer using individuals from the same three test folds matched to each of the three 301-Gene Variformer replicates.

Thus, any driver SNP, HSV, and control may have up to three standardized gradient attributions, and we averaged these for each model class. Error bars in Additional file 1: Fig. S10 represent 95% confidence intervals calculated using seaborn (v0.13.2) by performing 1000 bootstraps of SNPs from the driver/HSV and control groups after averaging.

## Supplementary Information

Additional file 1. Additional Figures S1 - S16.

## Data Availability

Code to train Variformer models and evaluate Variformer and Enformer models are available at https://github.com/shirondru/enformer_fine_tuning under a MIT license. A version of the code that is fixed at the time of writing is available at Zenodo [42]: 10.5281/zenodo.16969872. GTEx WGS [43] and ROSMAP [44] data are available from dbGaP (accession: phs000424.v9.p2) and Synapse AMP-AD Data Portal (accession: syn2580853), respectively.  Variformer model weights are available by request from the authors to researchers holding an active dbGaP Data Use Agreement for GTEx WGS data. Requests should be emailed to katherine.pollard@gladstone.ucsf.edu and must include documentation of an active dbGaP project for GTEx WGS data.
