# Supplementary material for: Deep-learning prediction of gene expression from personal genomes
Source: Genome Biol. 2026 Jan 6;27:19. doi: 10.1186/s13059-025-03926-7 (PMC12869966; doi:10.1186/s13059-025-03926-7)
Supplement: Supplementary file 1 — Additional file 1. Additional Figures S1 - S16. [file 13059_2025_3926_MOESM1_ESM.pdf]

ADDITIONAL FILE 1: SUPPLEMENTARY FIGURES

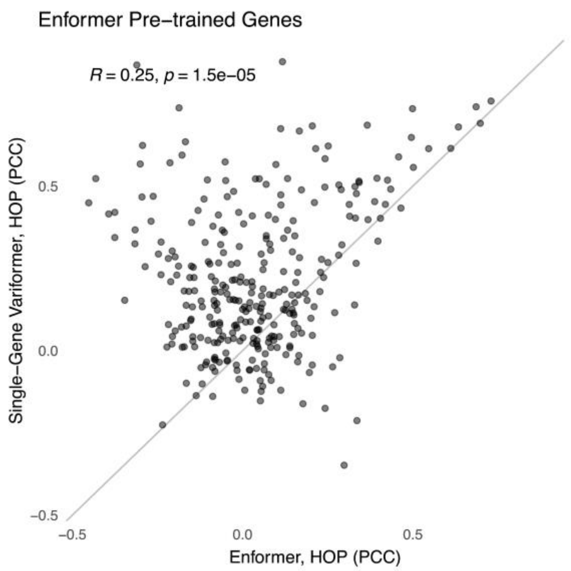

Figure S1: Fine-tuning improves predictions of expression variability.

PCC of Variformer trained on single genes (y-axis) versus Enformer (x-axis), both evaluated on HOP for each of the 301 training genes in Whole Blood. For most genes, Variformer explains more expression variability and is negatively correlated with observed expression less often. R^2 and PCC values are averaged as in (**Fig. 1B**).

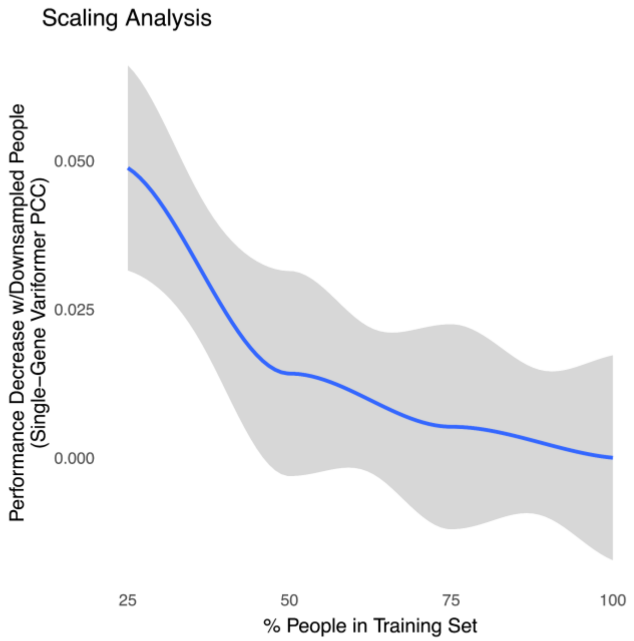

Figure S2: Fine-tuning performance while downsampling donors used to train.

Variformer models were trained on blood RNA-seq of 40 genes after downsampling the 536 train set individuals (**Methods**). Y-axis represents the decrease in PCC after using 25%, 50%, or 75% of individuals, relative to when training with 100% of the training set. PCC values are averaged over three model replicates for each downsampling group as in (**Fig. 1B**) We observe performance with 50% of the training set (~250 individuals) is comparable to performance after training on the full set, and although the trend suggests performance may further increase if more individuals were available. Models are evaluated on HOP, using the full set of test individuals. Grey area represents the 95% confidence interval of a LOESS curve fit across genes.

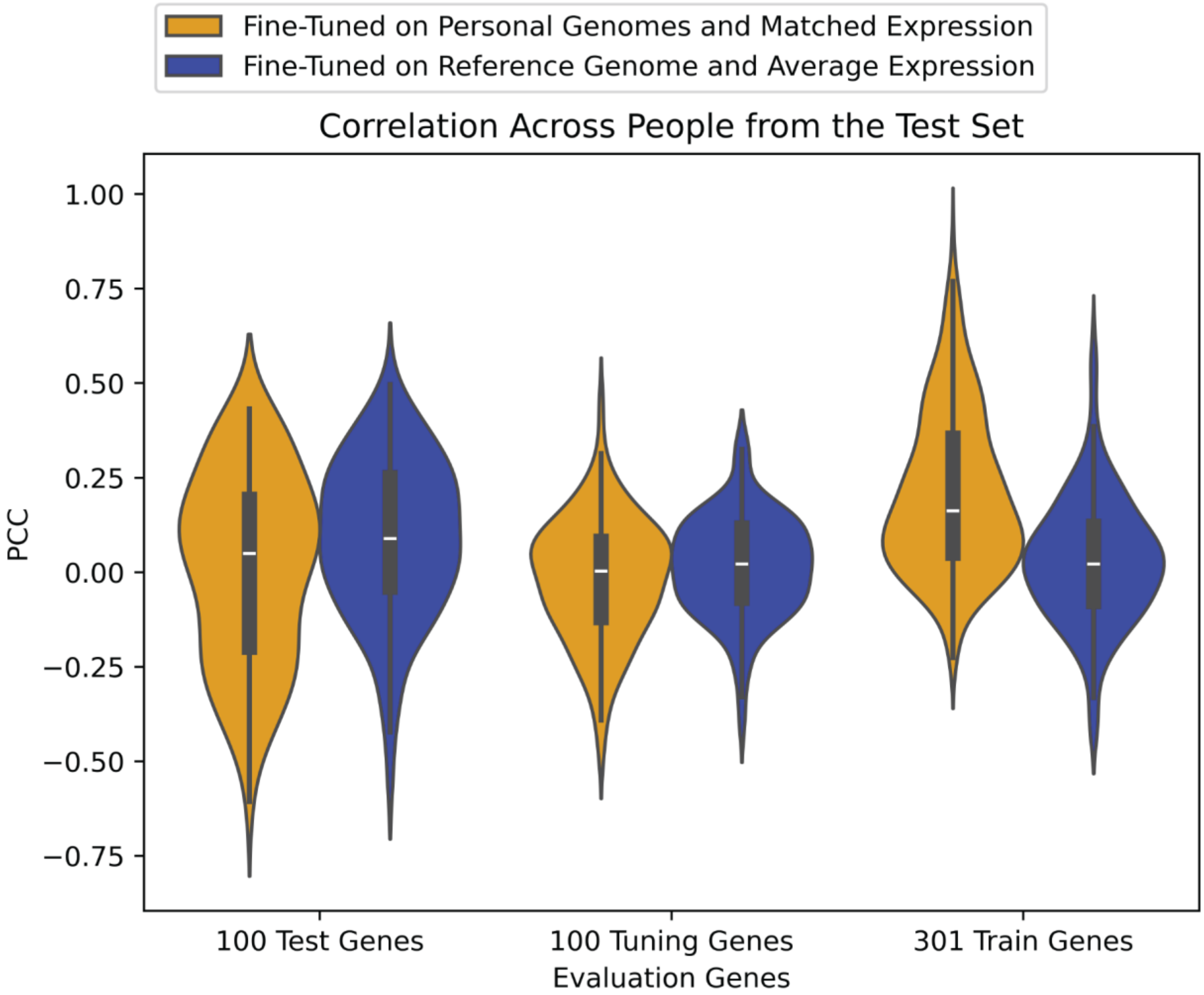

Figure S3: Comparison of Variformer models fine-tuned on personal genomes and matched expression versus the reference genome and population-averaged expression.

PCC of 301-Gene Variformer models, evaluated across individuals in the test set, using train genes (HOP) as well as unseen genes from the tuning or test sets (HOGP). Orange distributions represent Variformer models fine-tuned on personal genomes and matched gene expression values from GTEx. Blue distributions represent Variformer models fine-tuned using human reference genome (hg38) sequences and gene expression values averaged across individuals in the train set (**Methods**). Both models are evaluated using personal genomes and matched expression values.

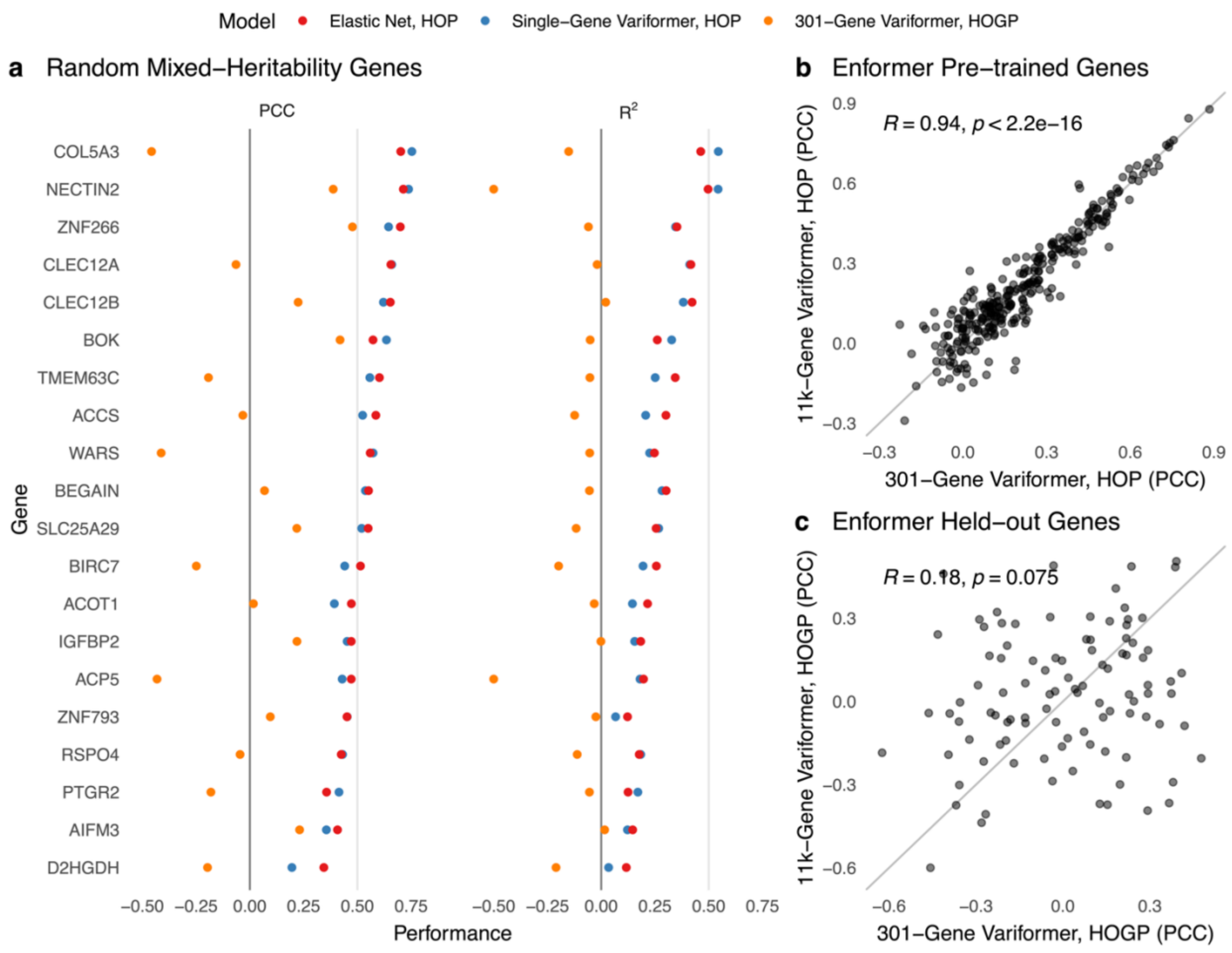

Figure S4: **Performance of multi-gene Variformer models on Train and Test Genes.**

Each point represents performance of a model evaluated on a single gene using GTEx Whole Blood expression values. Results are not averaged over replicates as in (**Fig. 1B**); they come from one model and one evaluation set only.

a) R^2 and PCC of elastic net (red) and single-gene Variformer models (blue) that were trained individually on 100 genes in the test set and evaluated on these same genes and HOP. Variformer models trained jointly on 301 train genes and then evaluated on HOGP using these 100 test genes (orange) underperform on the 100 unseen genes relative to the models trained directly on those genes. This panel shows 20/100 representative test genes with a range of cis-heritabilities. 301-gene Variformer occasionally exhibits strong PCC yet poor R^2 on these HOGP because it ranks individuals well but predicts much smaller than observed gene expression changes.

b) PCC of a Variformer model trained jointly on 301 genes and evaluated on each of these genes using HOP (x-axis) and of a Variformer model trained jointly on 11,429 genes (including the same 301 genes) and evaluated in the same way on the same genes. Each point represents one of the 301 genes used to train both models. Training on more genes did not improve performance on training genes.

c) PCC of the same multi-gene models as in (b) evaluated on HOGP using 100 test genes. Each point represents one test gene that was held-out from training of both models. Training on more genes did not improve performance on unseen genes.

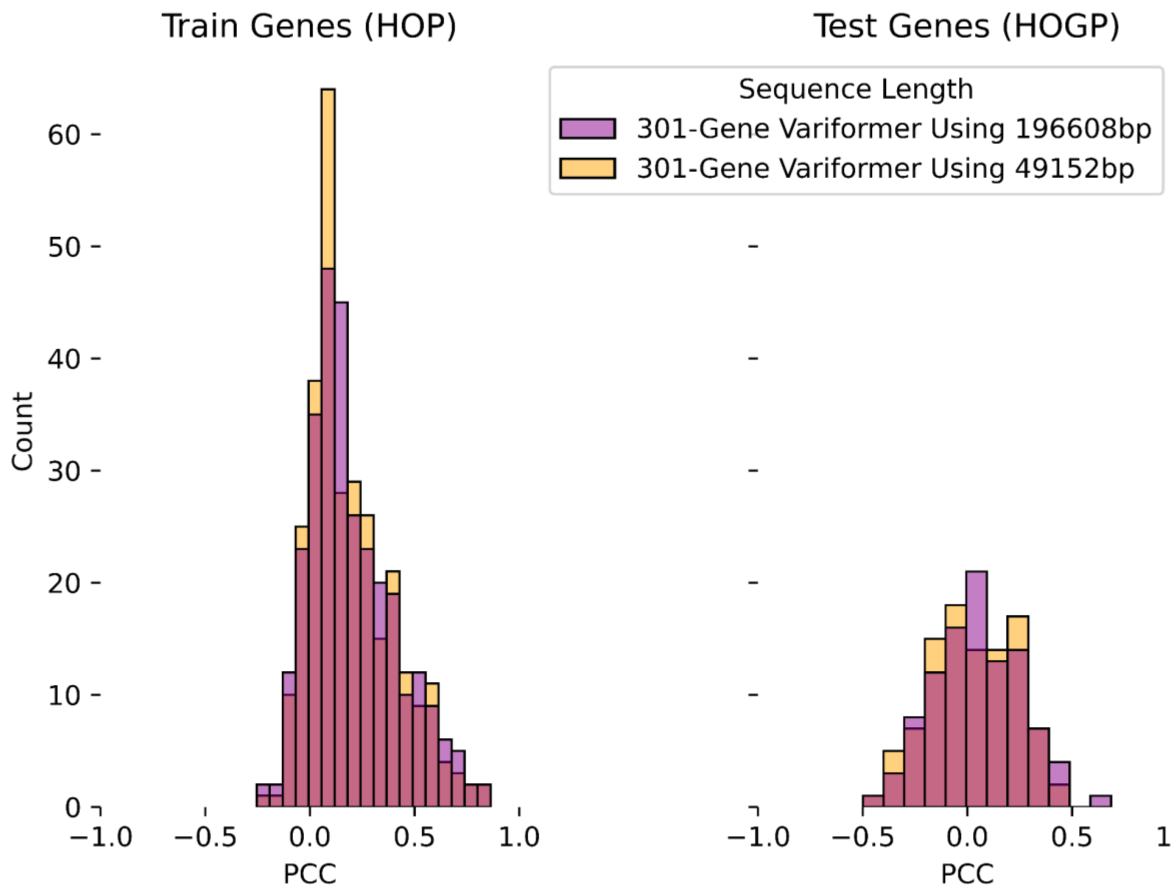

Figure S5: **Fine-tuning on longer sequences does not enable better cross-individual performance**. PCC of Variformer models trained jointly on 301 genes using GTEx Whole Blood data and either 196-kb DNA sequences (orange) or 49-kb sequences (blue) centered on each gene's TSS, evaluated on HOP using the same 301 train genes (left) or HOGP using 100 test genes (right). PCC values are averaged over three model replicates, as in (**Fig. 1B**).

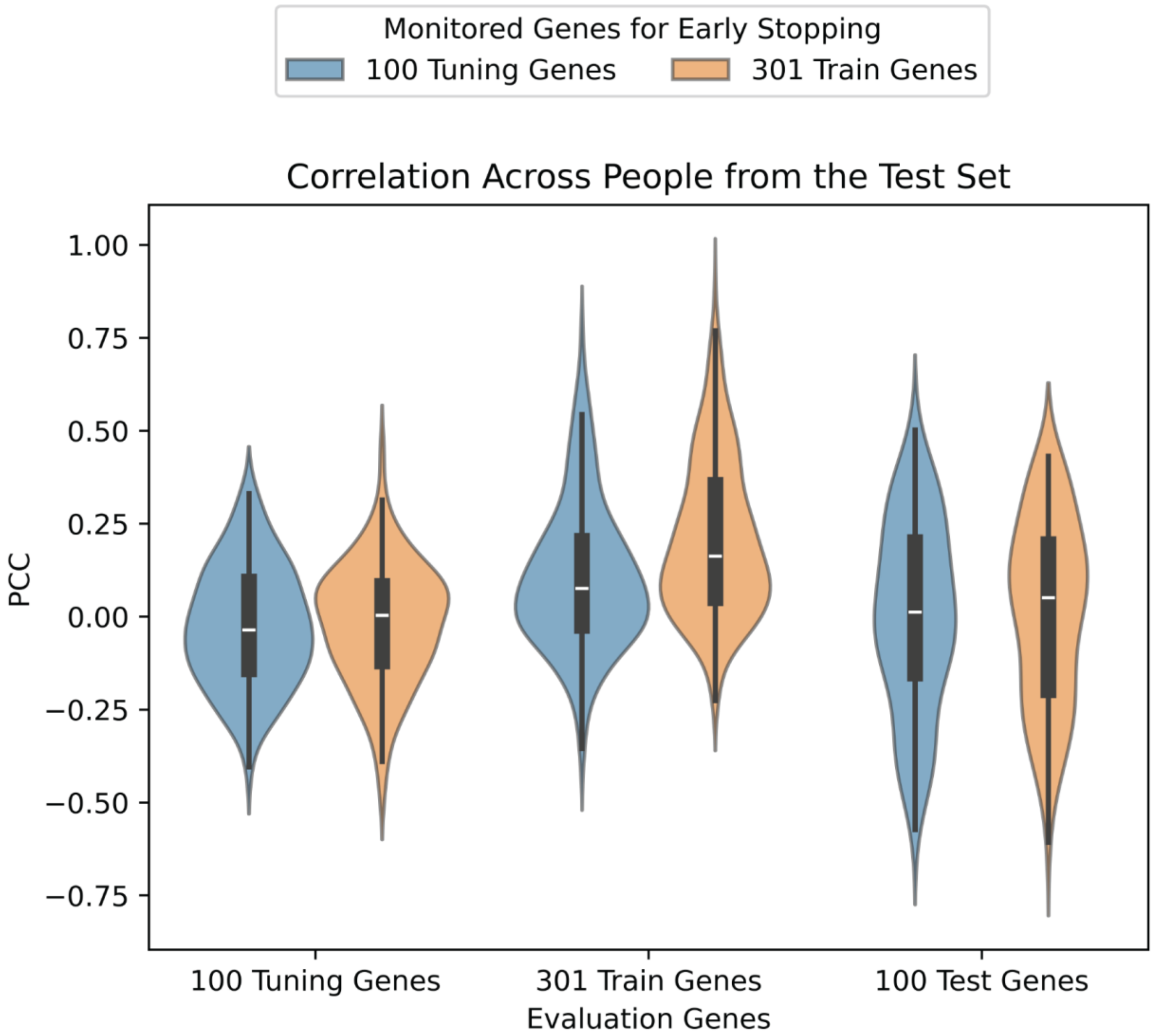

Figure S6: Comparison of performance on HOP and HOGP when using different gene sets for early stopping and checkpointing.

Cross-individual PCC among HOP or HOGP (X-axis; similar to **Additional file 1: Fig. S3**) when monitoring train genes (orange) or held-out genes (blue) for early stopping and checkpointing, evaluated using individuals from the test set.

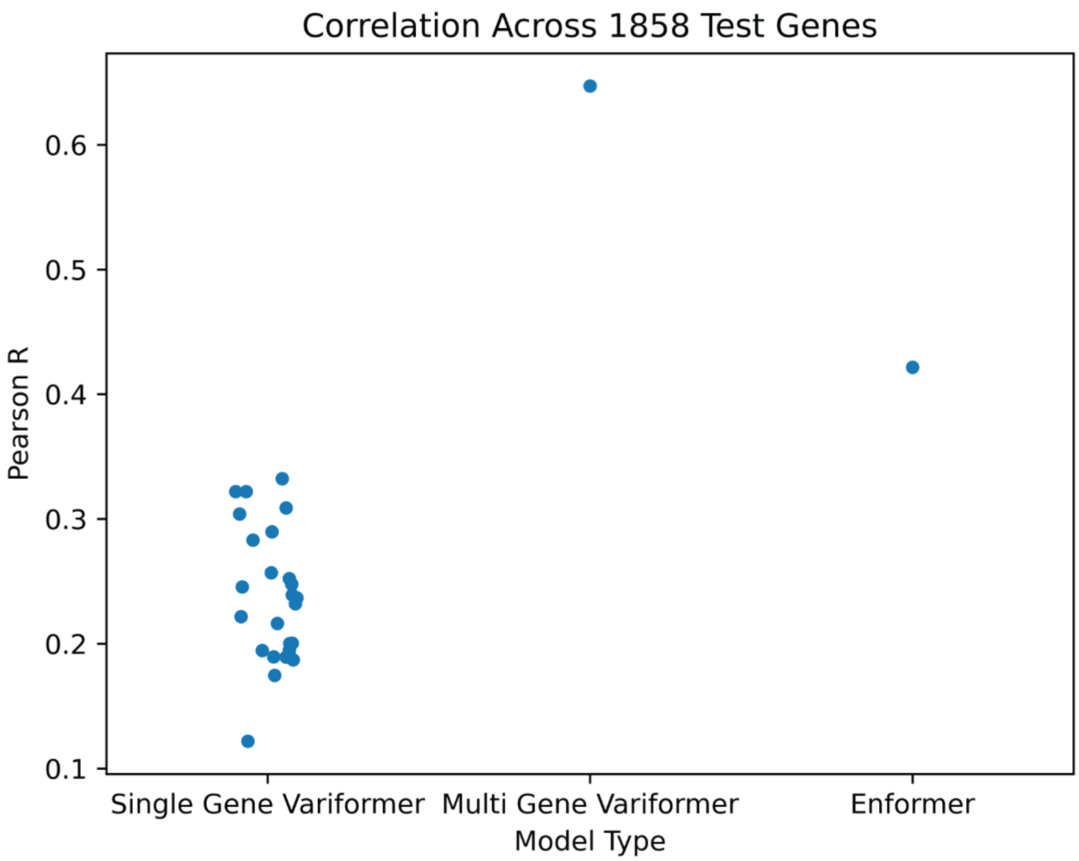

Figure S7: Evaluation of Variformer and Enformer on predicting population-averaged expression of unseen genes.

The correlation between predicted and observed expression of 1858 genes in Enformer and Variformer's test sets. The human reference genome (hg38) was passed in as input and the observed values were defined as gene expression values averaged among all GTEx individuals. We note that Enformer is disadvantaged relative to Variformer, because Variformer was directly fine-tuned on GTEx TPM values (**Methods**).

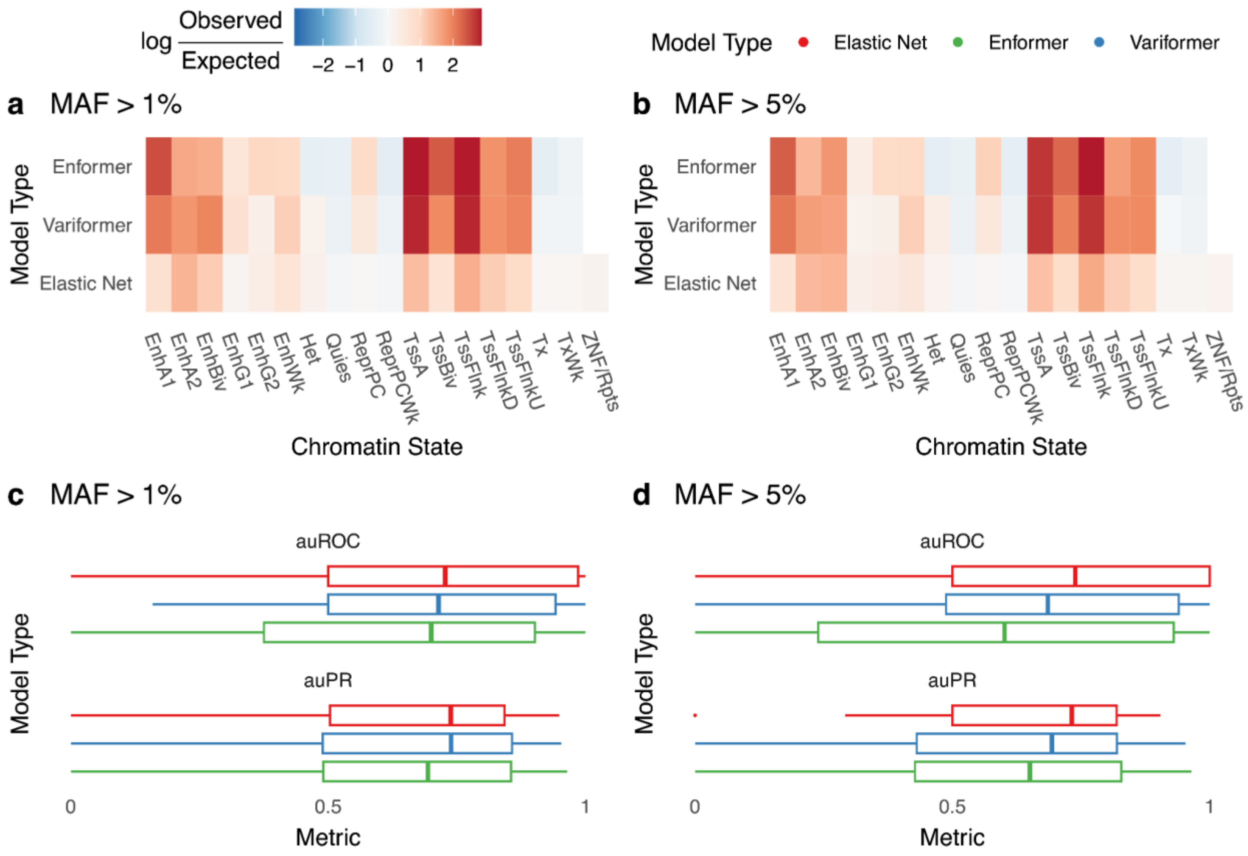

Figure S8: Functional signatures after removing rare variants.

a) Enrichment of HSVs with minor allele frequency (MAF) >1% across ChromHMM chromatin states (as in **Fig. 3A**).

b) Enrichment of HSVs with minor allele frequency (MAF) >5% across ChromHMM chromatin states (as in **Fig. 3A**).

c) Distribution of area under the Receiver Operating Characteristic (auROC) and Precision-Recall (auPR) curves for predicting fine-mapped Whole Blood GTEx eQTLs (as in **Fig. 3C**) for variants with MAF >1%.

d) Distribution of area under the Receiver Operating Characteristic (auROC) and Precision-Recall (auPR) curves for predicting fine-mapped Whole Blood GTEx eQTLs (as in **Fig. 3C**) for variants with MAF >5%.

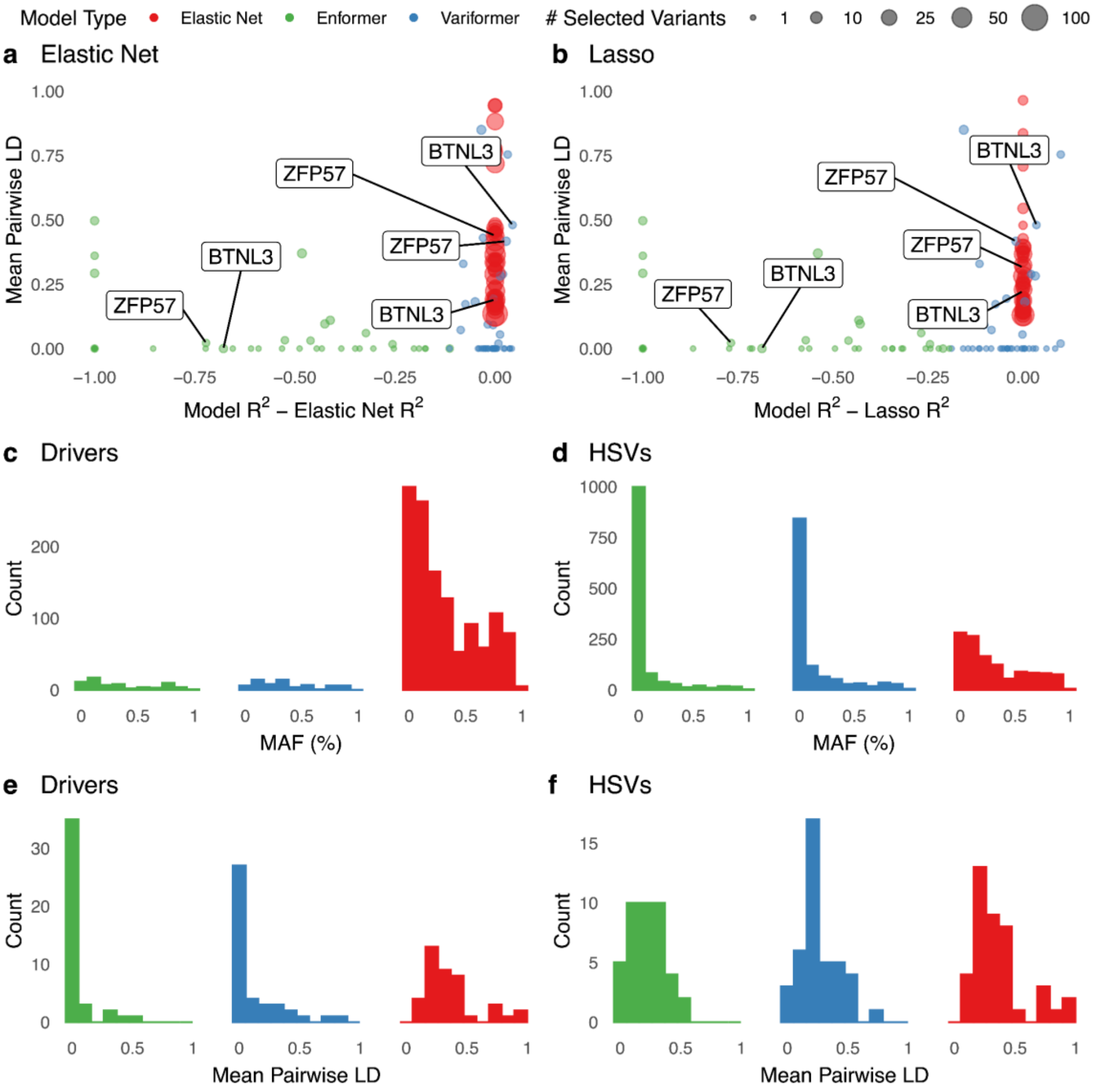

Figure S9: Properties of driver variants.

All panels analyzed on 42/301 genes with Variformer $R^2 > 0.2$ in Whole Blood**.**

a) Model performance, evaluated on HOP, relative to elastic net plotted against the mean pairwise linkage disequilibrium (LD) of selected variants, with the number of selected variants (Enformer/Variformer: drivers (**Methods**), elastic net: non-zero coefficients) indicated by size. Identical to **Fig. 4E**.

b) Same as (**A**) but fitting a lasso linear model.

c) Distribution of minor allele frequency (MAF) for Enformer and Variformer driver variants, and elastic net non-zero coefficients.

d) Distribution of minor allele frequency (MAF) for all Enformer and Variformer HSVs, and elastic net non-zero coefficients.

e) Mean pairwise LD of Enformer and Variformer driver variants and elastic net non-zero coefficients.

f) Mean pairwise LD of all Enformer and Variformer HSVs and elastic net non-zero coefficients.

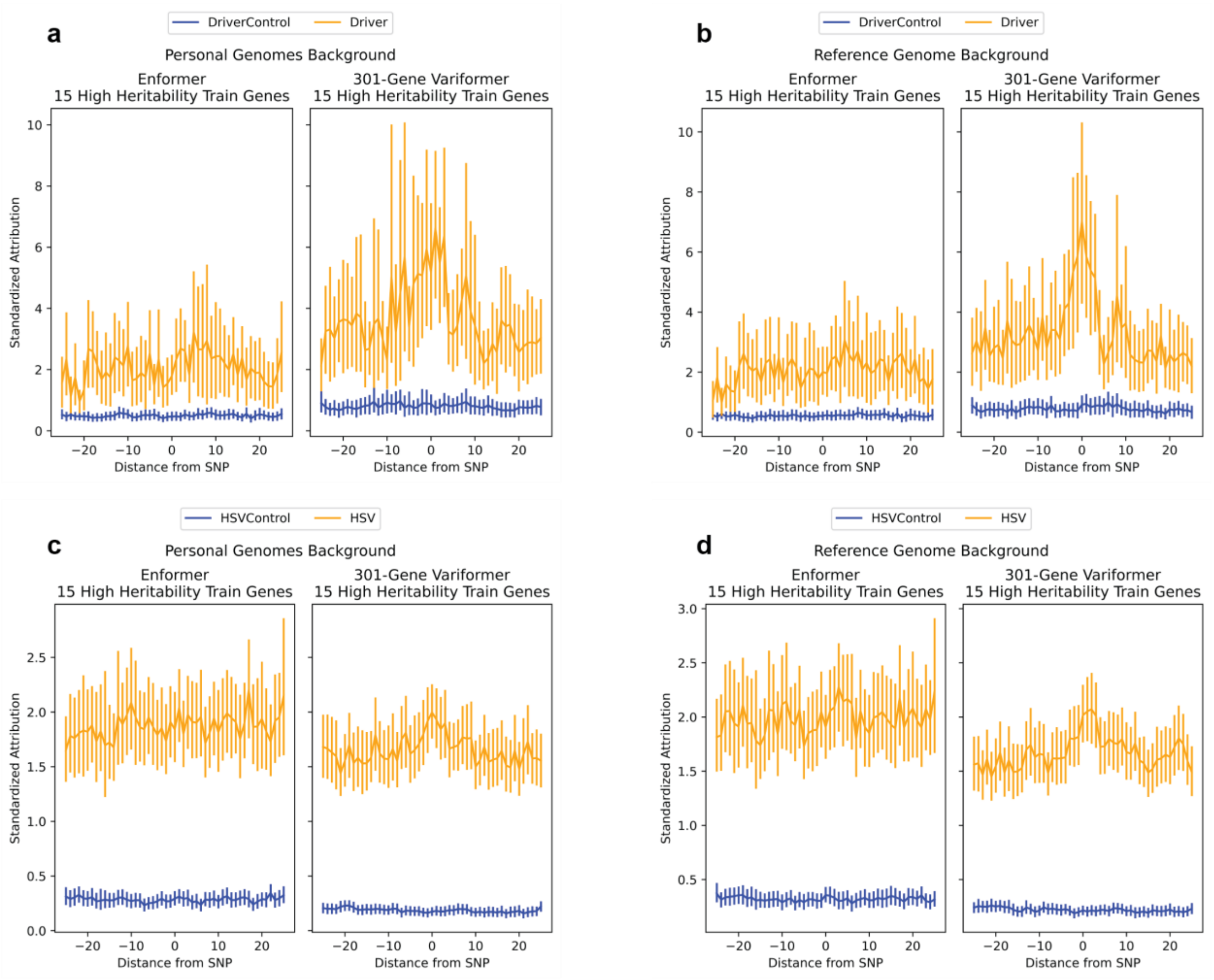

Figure S10: Comparison of input gradient attributions for model-prioritized and control SNPs. Comparison of standardized gradient attributions (**Methods**) between driver SNPs (**A & B**) or HSVs (**C & D**) utilized by Enformer (left) or 301-gene Variformer (right) compared to control SNPs matched to be of a similar minor allele frequency, a similar distance from the TSS, and around the same gene as drivers/HSVs. Standardized attributions at the focal SNP position are centered to appear at x = 0, and the attributions in the surrounding 50bp window are also shown. Standardized gradient attributions come from personal genomes using individuals from the test set (**A** & **C**) or the human reference genome (hg38; **B** & **D**). For each driver SNP, HSV, and control, we averaged the standardized attribution coming from each model. Standardized gradient attributions for each SNP were averaged over three replicates **(Methods**) and error bars represent 95% confidence intervals calculated by bootstrapping the driver/HSV and control groups.

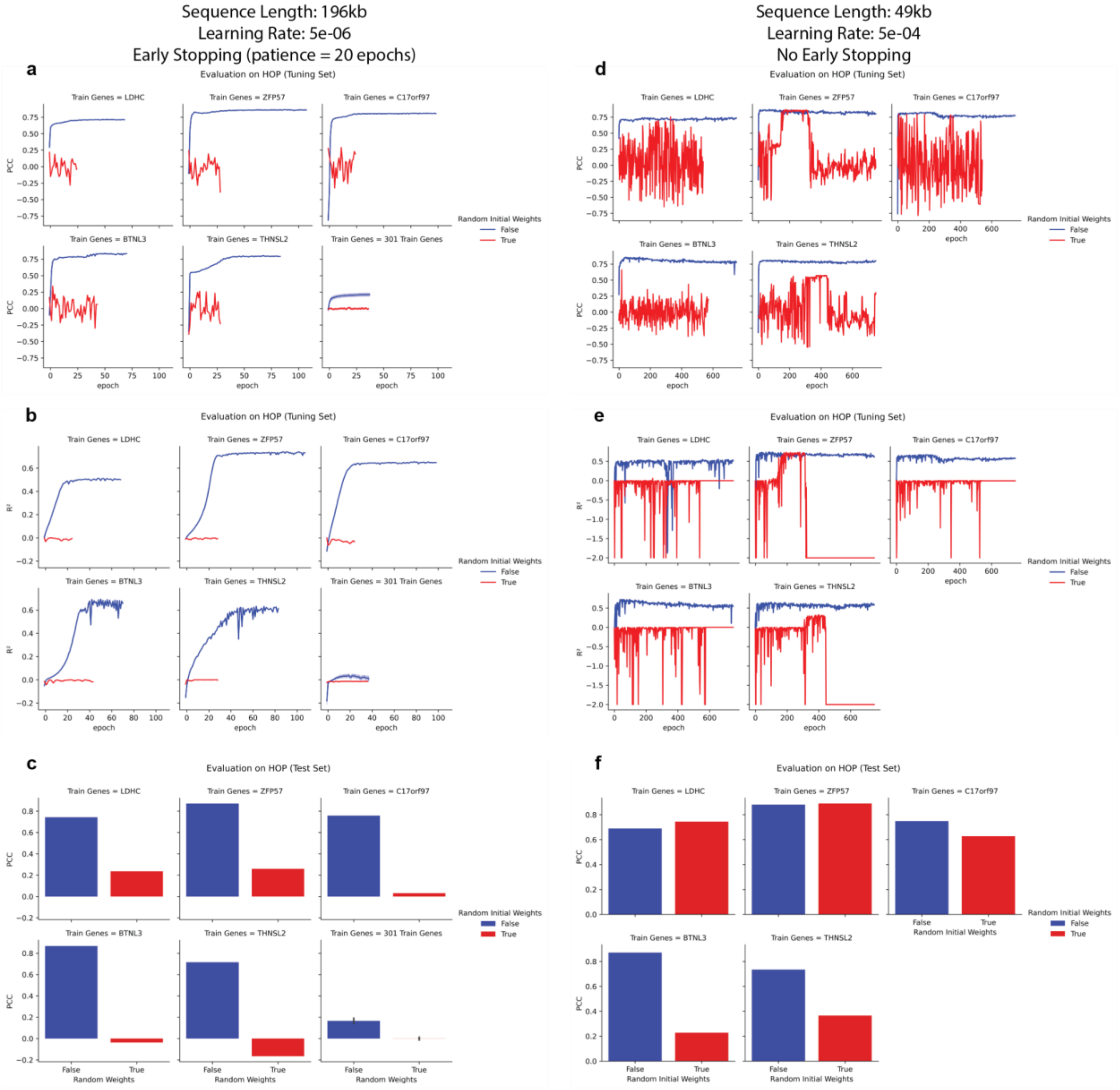

Figure S11: Comparison of fine-tuned versus randomly initialized Variformer models.

PCC (**A**) or R^2 (**B**) achieved by Variformer models that were fine-tuned starting with Enformer's pre-trained weights (blue) or with the same architecture and hyperparameters but random weights (red). PCC/R^2 metrics were evaluated on HOP in the tuning set during training. (**C**) Final performance on HOP of the Variformer models in (**A**) and (**B**) evaluated on individuals in the test set. (**D-F**) Same as (**A-C**) but using a greater learning rate, shorter sequence (5e-04 & 49kb vs 5e-06 & 196kb), and disabling early stopping. These hyperparameters helped improve performance in some cases when training from

random initial weights, although performance during training remained more stochastic. This suggests training from random initial weights is possible in principle but is more challenging, requiring additional hyperparameter tuning and more compute resources. We note that training from random weights selects a mostly disjoint variant set from pretrained Enformer. Combined with its lower performance, this suggests training from random weights on limited data learns inferior regulatory grammar. R^2 values are clipped at -2 for axis visibility.

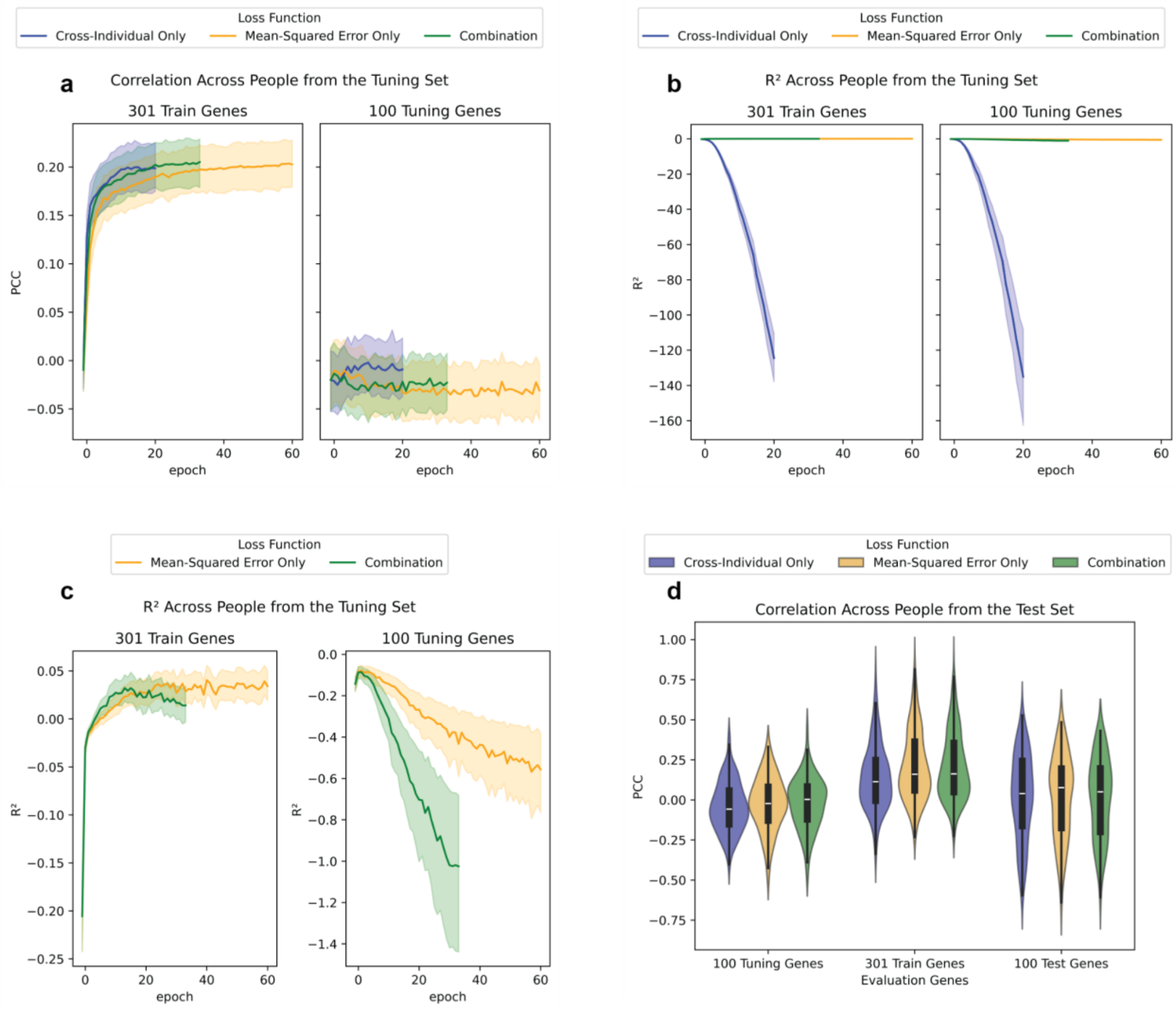

Figure S12: Comparison of performance on HOP and HOGP with different loss functions. Cross-individual PCC (**A**) or R^2 (**B & C**) during training of 301-Gene Variformer models, evaluated on HOP (left) or HOGP (right) from the tuning set, after training with different loss functions. Our loss function included equal contributions from two components, a mean-squared error component and a component that emphasized cross-individual differences (**Methods**). Performance from our original loss function is shown in green, while performance using only one of the two individual components is shown in blue and yellow. While the choice in the loss function does not affect HOGP results, incorporating the cross individual component leads to quicker convergence on HOP. When using only the cross-individual loss function, Variformer learns to predict expression differences from HOP (**A**) but cannot accurately predict the scale of these expression differences, leading to strong negative R^2 values (**B**); (**C**) shows R^2 values

after this result was removed to increase axis visibility. Cross-individual PCC from the same models in (**A-C**), evaluated on HOP or HOGP (X-axis) using individuals from the test set (**D**).

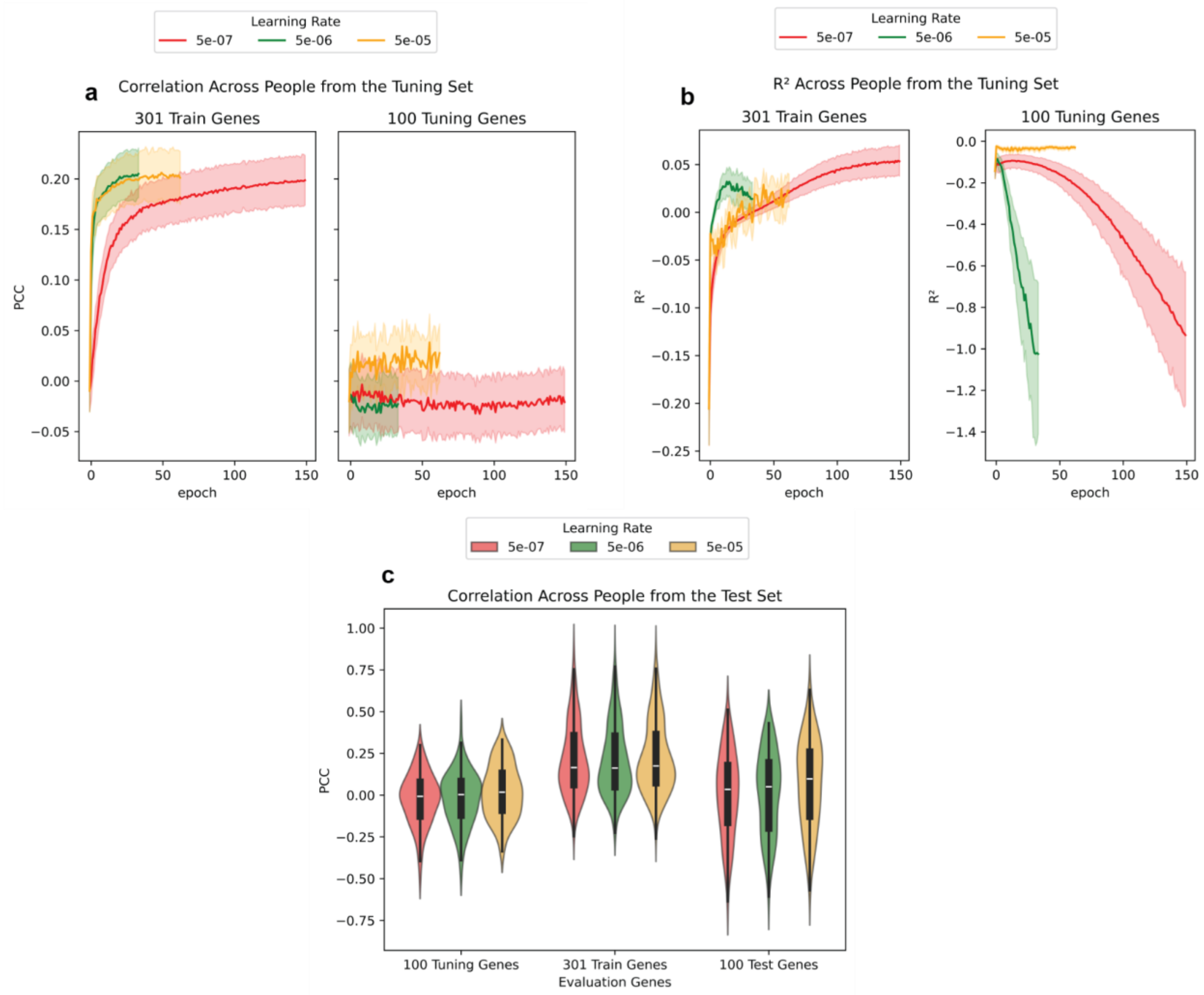

Figure S13: Comparison of performance on HOP and HOGP with different learning rates.

Cross-individual PCC (**A**) or R^2 (**B**) during training of 301-Gene Variformer models, evaluated on HOP (left) or HOGP (right) from the tuning set, after training with different learning rates. Cross-individual PCC from the same models in (**A & B**), evaluated on HOP or HOGP (X-axis) using individuals from the test set (**C**). A learning rate of 5e-06 was used for fine-tuning throughout the rest of this manuscript.

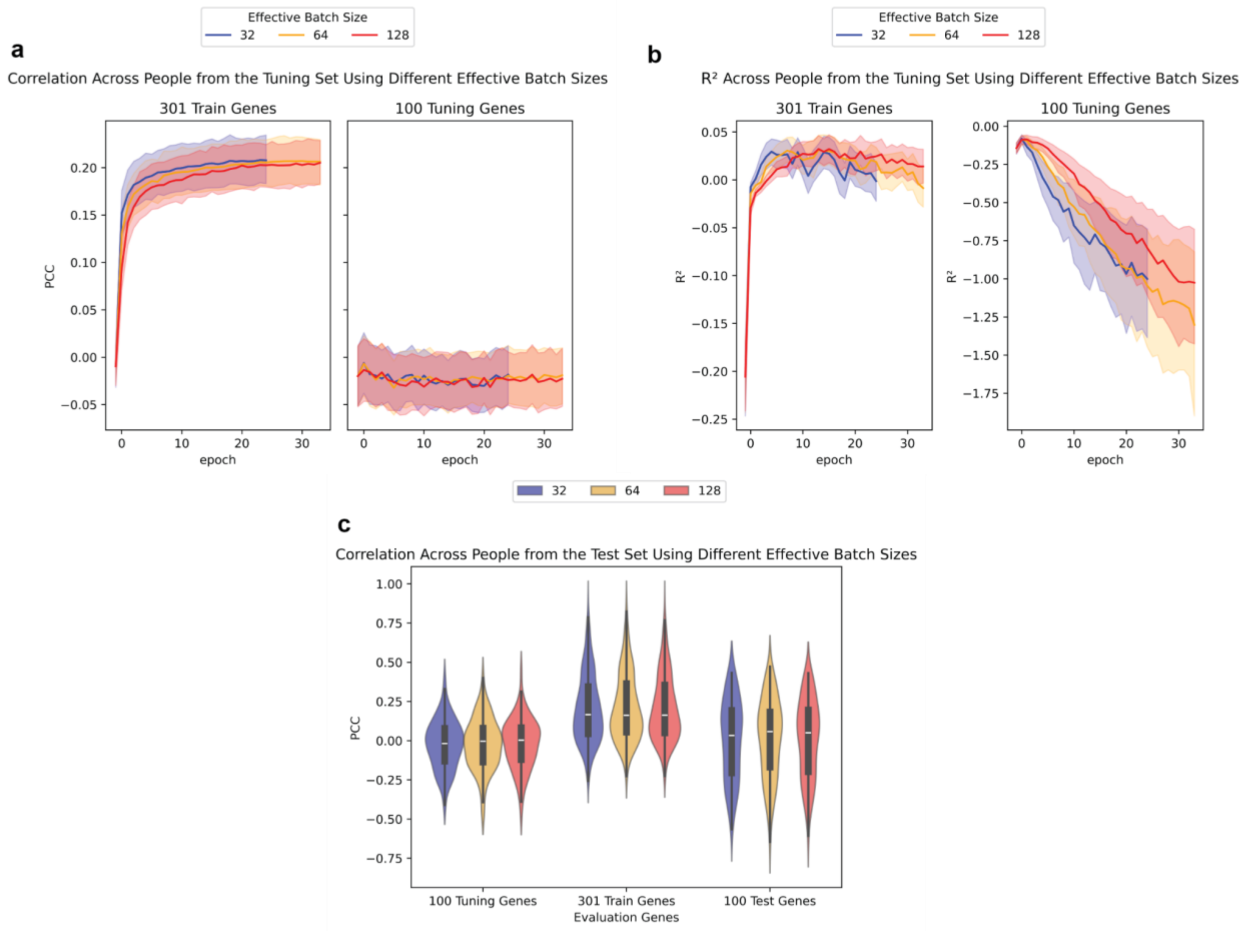

Figure S14: Comparison of performance on HOP and HOGP with different effective batch sizes using gradient accumulation.

Cross-individual PCC (**A**) or R^2 (**B**) during training of 301-Gene Variformer models, evaluated on HOP (left) or HOGP (right) from the tuning set, after training with different effective batch sizes using gradient accumulation. Cross-individual PCC from the same models in (**A & B**), evaluated on HOP or HOGP (X-axis) using individuals from the test set (**C**). Our training batch size was 32, and we accumulated gradients for four batches to achieve an effective batch size of 128 throughout the rest of this manuscript (red). We also attempted to accumulate gradients for one batch (i.e, no gradient accumulation; blue) or two batches (yellow). These results suggest decisions related to gradient accumulation do not have a strong impact on training personal genome predictors.

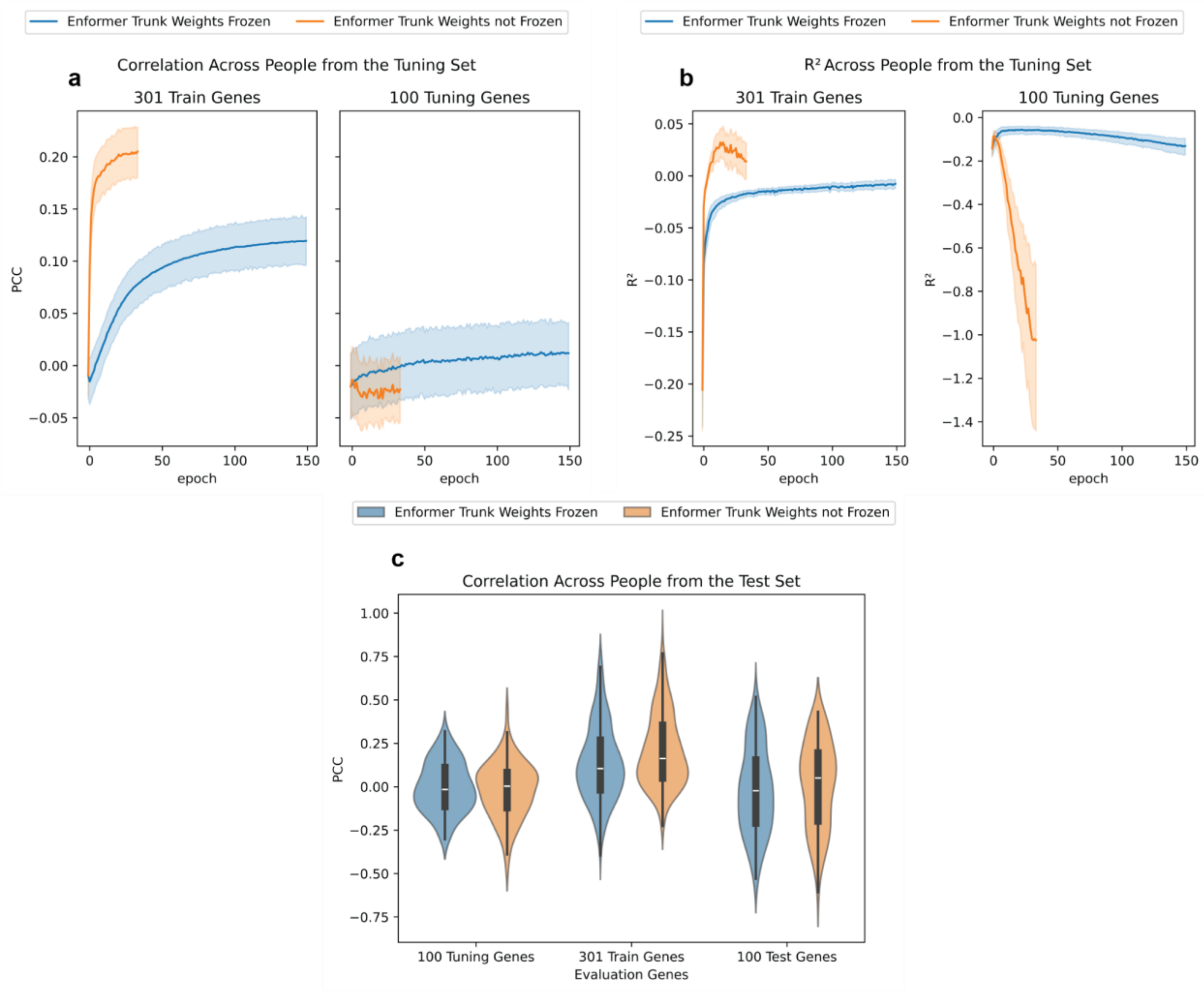

Figure S15: Comparison of performance on HOP and HOGP with and without freezing Enformer's weights.

Cross-individual PCC (**A**) or R^2 (**B**) during training of 301-Gene Variformer models, evaluated on HOP (left) or HOGP (right) from the tuning set, after training with and without keeping Enformer trunk weights (i.e, all of Enformer's weights except for the output heads) frozen. Cross-individual PCC from the same models in (**A & B**), evaluated on HOP or HOGP (X-axis) using individuals from the test set (**C**).

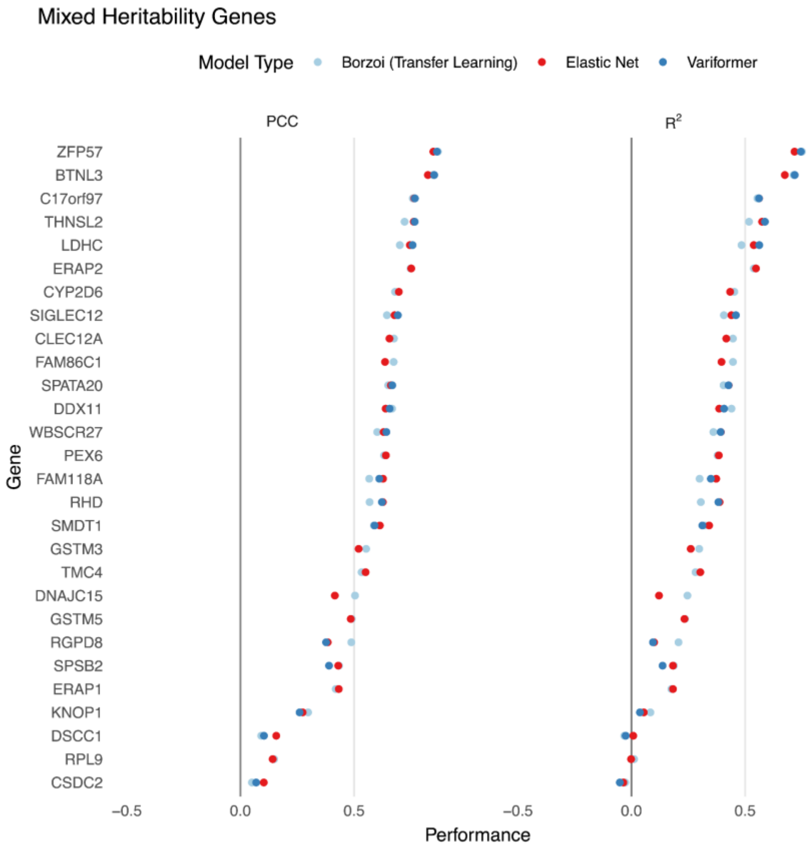

Figure S16: Performance of Borzoi after transfer learning.

R^2 and PCC of models trained on single genes using GTEx Whole Blood data. All models were trained on single genes and evaluated on HOP. Results come from one model trained on each gene. Elastic net, fine-tuned Enformer (Variformer), and Borzoi[26] models after transfer learning (model weights frozen, output layer trained) perform comparably. We did not evaluate the published Borzoi model without transfer learning, but we note that it outperformed the published Enformer model on other benchmarks[26].
